# Does acute caffeine ingestion improve high-intensity interval exercise performance? A systematic review and meta-analysis

**DOI:** 10.3389/fphys.2026.1858094

**Published:** 2026-05-21

**Authors:** Yixiang Peng, Lei He, Wenxin Du, Baiyu Liu, Xinkai Wang, Yun Gong

**Affiliations:** 1 Faculty of Health Sciences and Sports, Macao Polytechnic University, Macao SAR, China; 2 Department of Physical Education, College of Education, Zhejiang University, Hangzhou, Zhejiang, China; 3 School of Physical Education, Shanghai Normal University, Shanghai, China; 4 School of Athletic Performance, Shanghai University of Sport, Shanghai, China

**Keywords:** caffeine, ergogenic aids, exercise performance, high−intensity interval exercise, intermittent sports

## Abstract

**Objective:**

This systematic review and meta-analysis evaluated the effects of caffeine (CAF) on high−intensity interval exercise (HIIE) performance and examined potential moderators.

**Methods:**

Several databases were searched for studies of CAF on HIIE performance. Pooled effects were calculated using Hedge’s *g* (*g*) via a three-level random-effects meta-analysis. Subgroup analyses were performed based on sex, training status, CAF dose, CAF form, and interval type. A meta-regression analysis was conducted to investigate the potential moderating effect of the rest/work ratio on HIIE performance.

**Results:**

Twenty studies were included (n = 320; 57 females). CAF significantly improved HIIE performance (*g* = 0.28, 95% CI = 0.14 to 0.43), concurrently elevating blood lactate (*g* = 0.50, 95% CI = 0.22 to 0.79) and glucose (*g* = 0.56, 95% CI = 0.17 to 0.96). Subgroup analyses demonstrated significant improvements across all sexes (*g* = 0.30–0.34), trained athletes (*g* = 0.44), CAF dose (low and moderate) (*g* = 0.28–0.33), CAF form (capsule and beverage) (*g* = 0.32–0.33), and HIIE protocol (repeated short sprints and short intervals) (*g* = 0.16–0.36), with no subgroup differences (all *p* > 0.05). The rest/work ratio was a significant moderator of HIIE performance (*β_2_* = 0.003, *p* = 0.017).

**Conclusion:**

CAF ingestion significantly enhances HIIE performance, with ergogenic benefits observed across both sexes and trained athletes. Effective ergogenic benefits can be achieved with a low dose (~3 mg/kg), administered as a capsule or beverage. Notably, meta-regression indicates that the rest/work ratio is a critical moderator of HIIE performance, with evidence of a nonlinear association.

## 1 Introduction

High-intensity interval exercise (HIIE) refers to repeated bouts of near-maximal to “all-out” effort interspersed with recovery periods of low-intensity work or rest (Coates et al., 2023). The capacity to perform HIIE is a critical determinant of performance in intermittent sports (e.g., team, racket, and combat sports) (Buchan et al., 2013; Salinero et al., 2019; Diaz-Lara et al., 2023), as high-intensity actions (e.g., jumping, tackling, and repeated approach spike jumps) directly influence scoring opportunities and can ultimately influence match outcomes (Spencer et al., 2005). However, HIIE performance progressively declines over time due to central fatigue (e.g., adenosine accumulation) and peripheral mechanisms (e.g., phosphocreatine depletion) (Nehlig et al., 1992; Gaitanos et al., 1993). To optimize athletic performance, effective nutritional strategies are required to sustain HIIE performance, ultimately translating to enhanced competitive match-play.

Caffeine (CAF) is one of the most commonly used ergogenic aids among athletes across various sports (Del Coso et al., 2011). Its efficacy in enhancing endurance (Southward et al., 2018; Chen et al., 2024) and resistance (Grgic and Del Coso, 2021; Xiao et al., 2025) exercise performance is well-established. In recent years, increasing attention has focused on the effects of CAF on HIIE performance. However, given that HIIE encompasses a highly diverse and complex range of protocols (Tschakert and Hofmann, 2013), findings in this area remain inconclusive. For instance, a previous systematic review by Lopes-Silva et al. (2019) concluded that CAF ingestion fails to enhance repeated sprint ability (RSA, a form of HIIE). Nevertheless, this evidence may not be generalizable to other HIIE modalities and is further constrained by several methodological limitations. First, their analysis was limited by incomplete study inclusion. The omission of three relevant trials could potentially alter their pooled effect estimate (Paton et al., 2001; Del Coso et al., 2012; Lara et al., 2014). For example, Lara et al. (2014) reported that 3 mg/kg of CAF significantly enhanced running speed during the sprint test (24.2 ± 1.6 vs 24.5 ± 1.7 km/h; P < 0.05). Second, the previous review did not investigate whether participant characteristics, supplementation strategies, and HIIE protocols moderate the effects of CAF on HIIE. This gap is particularly noteworthy given that primary studies suggest greater benefits are often observed in males, trained athletes, moderate doses (4–6 mg/kg), and critically, under specific interval designs such as varying rest/work ratios (Carrillo and Benitez, 1996; Davis and Green, 2009; Fiorenza et al., 2019; Khodadadi et al., 2025; Xue et al., 2025). Consequently, a comprehensive meta-analysis is warranted to systematically evaluate the effects of CAF on HIIE performance and to examine these potential moderating factors.

Therefore, this study used a three-level meta-analysis to evaluate the effects of CAF on HIIE while accounting for dependence among multiple performance outcomes reported within the same study. Meta-regression was further performed to examine the moderating role of the rest/work ratio, providing a more nuanced understanding of CAF’s ergogenic effects and their practical implications for intermittent sports nutrition.

## 2 Methods

The systematic review adhered to the 2020 Preferred Reporting Items for Systematic Reviews and Meta-Analyses (PRISMA) statement (Page et al., 2021). Additionally, this review was registered in the International Prospective Register of Systematic Reviews (PROSPERO) database (CRD420261337845).

### 2.1 Literature search

The literature review was conducted across PubMed, Web of Science, Cochrane Database, and Scopus from inception to October 18, 2025. We used combinations of subject headings and keywords with Boolean operators (“AND,” “OR,” and “NOT”) and truncation, including terms such as “CAF”, “coffee”, “energy drink”, “high-intensity interval exercise”, “interval exercise” (detailed search strategy is presented in **Supplementary Appendix S1**). Additionally, searches of PROSPERO of Systematic Reviews were conducted to determine whether protocols for related systematic reviews had already been published. Finally, to avoid missing any relevant literature, we also conducted hand searching in reference citations of identified reviews.

### 2.2 Selection process

Deduplication of retrieved records was performed manually by an independent reviewer (P.Y.) using EndNote X9 (Clarivate Analytics, Philadelphia, PA, USA). Then, two independent researchers (P.Y. and H.L.) screened the titles and abstracts. If consensus could not be reached, a third independent researcher (L.B.) was consulted. Finally, two researchers (P.Y. and H.L.) independently reviewed the full texts of selected articles for final inclusion. Any discrepancies were resolved based on the predefined inclusion and exclusion criteria.

### 2.3 Selection criteria

A set of *a priori* inclusion and exclusion criteria were used to evaluate study eligibility according to the PICOS framework: (1) P: healthy adult human participants (≥ 18 years of age); (2) I: studies examining the effects of CAF ingestion on HIIE performance; (3) C: included a placebo group as the comparator; (4) O: eligible studies reporting at least one outcome of overall HIIE performance [i.e., fastest time, average time, total time, mean power output (MPO), peak power output (PPO), and total work] or physiological responses [i.e., heart rate (HR), blood lactate concentration (BLC), rating of perceived exertion (RPE), glucose]; (5) S: utilized a double-blind randomized crossover design.

Exclusion criteria were: (1) non-English articles; (2) studies examining long-term CAF interventions during HIIE; (3) systematic reviews or meta-analyses, case studies, non-peer-reviewed manuscripts, and conference proceedings; (4) animal studies; (5) studies on therapeutic or disease-related outcomes.

### 2.4 Assessment of methodological quality

Risk of bias was assessed using the Cochrane Risk of Bias Assessment Tool via Review Manager 5.4 software (The Cochrane Collaboration, 2020; Cohen, 1988). The tool evaluates (1) random sequence generation; (2) allocation concealment; (3) blinding of participants and personnel; (4) blinding of outcome assessment; (5) incomplete outcome data; (6) selective reporting; (7) other bias. Ratings for each category are denoted as either “low risk” (“+”), “high risk” (“-”) or “unclear risk” (“?”). Quality assessment was performed independently by two investigators (P.Y. and H.L.), with discrepancies resolved by discussion or through consultation with a third reviewer (L.B.).

Additionally, the physiotherapy evidence database (PEDro) (de Morton, 2009) scale was used to assess the risk of bias and methodological quality of included studies. Studies were scored on a scale of 0–10, with scores ≥ 6 indicating high quality, 4–5 moderate quality, and ≤ 3 low quality.

### 2.5 Data extraction and study coding

Data extraction was independently conducted by two researchers (P.Y. and W.X.) using Excel (Version 16.93, Microsoft, Redmond, WA, USA). Extracted data included author details, sample characteristics (e.g., number, sex, training status), exercise protocol, overall HIIE performance outcomes (e.g., MPO, PPO, total work, fastest time, average time, and total time), main physiological responses (e.g., HR, BLC, glucose) and RPE. Discrepancies in data extraction were solved by consensus. Relevant data were extracted using WebPlotDigitizer 4.7 (Drevon et al., 2017) if data were missing or presented only in graphical form. When a study reported multiple sets of target data, they were combined into a single mean value. We were converted standard errors (SE) to standard deviations (SD) using the formula recommended in the Cochrane guideline (Cumpston et al., 2019) if the study provide SE. Some studies were included multiple times in the analysis due to independent comparisons across different doses to ensure that data related to HIIE and CAF were fully analyzed.

### 2.6 Statistical analyses

#### 2.6.1 Calculation of effect size and variance

The effect of CAF ingestion on HIIE performance was analyzed by comparing CAF with the placebo condition. The mean difference (MD) and the SD of the change in means ( ${\text{SD}}_{\text{pooled}}$) were calculated according to the recommendations of the Cochrane handbook for the evaluation of intervention systems (version 6.5, 2024), using the following formula (Higgins et al., 2019). The first step involved calculating the difference in means:


$$\text{MD}=M_{\text{CAF}}-M_{\text{PLA}}$$

where $M_{\text{CAF}}$ is the reported mean values of CAF group and $M_{\text{PLA}}$ is the reported mean values of placebo group.

Then the ${\text{SD}}_{\text{pooled}}$ for crossover studies was calculated as follows (Hedges and Shymansky, 1989):


$${\text{SD}}_{\text{pooled}}=\sqrt{\frac{{\text{SD}}_{\text{CAF}}^{2}+{\text{SD}}_{\text{PLA}}^{2}}{2}}$$

where ${\text{SD}}_{\text{CAF}}$ is the SD from CAF group and ${\text{SD}}_{\text{PLA}}$ is the SD from placebo group.

Considering the relatively small sample sizes of most included studies, in each analysis we used Hedge’s *g* (*g*) as the point estimate of the mean effect size, which for crossover studies is as follows (Hedges, 1983):


$$\text{Hedge’s g}=\frac{M_{\text{CAF}}-M_{\text{PLA}}}{{\text{SD}}_{\text{pooled}}}\times (1-\frac{3}{4(N-1)-1})$$

where N is the total sample size. *g* was classified as *trivial* (SMD < 0.20), *small* (0.20 ≤ SMD < 0.50), *moderate* (0.50 ≤ SMD < 0.80), or *large* (SMD ≥ 0.80) (Cumpston et al., 2019).

For the crossover experimental design, the SE of g was calculated using the following formula (Hedges, 1983):


$$\text{SE}=\sqrt{\frac{1}{N}+\frac{g^{2}}{2N}}\times \sqrt{2(1-r)}$$

where r is the correlation coefficient between the CAF and placebo conditions. Few included studies reported the within-subject correlation coefficient (r). We also reviewed prior meta−analyses in this field, neither of which reported r values. Therefore, in accordance with the Cochrane Handbook (Cumpston et al., 2019), we assumed a correlation of r = 0.50 for the primary analysis. To assess the robustness of our findings, we conducted sensitivity analyses using alternative r values, specifically r = 0.20 (lower bound) and r = 0.80 (upper bound), for the overall HIIE performance outcomes.

#### 2.6.2 Meta-analysis and heterogeneity

Traditional two-level meta-analysis was conducted to aggregate the physiological response with the *meta* and *metafor* packages in R (V.4.2.0, R Core Team, Vienna, Austria) (Viechtbauer, 2010). For two-level meta-analysis, we used random-effects model and standardized mean differences (SMDs), which estimated as Hedges’ *g* using the restricted maximum likelihood method (REML) to ensure stability. Hedge’s *g* was classified as *trivial* (SMD < 0.20), *small* (0.20 ≤ SMD < 0.50), *moderate* (0.50 ≤ SMD < 0.80), or *large* (SMD ≥ 0.80) (Cumpston et al., 2019).

We also conducted a three-level meta-analysis following recommendations of Assink and Wibbelink (Assink and Wibbelink, 2016) to address the double counting or missed correlations in HIIE performance with nested or multiple effect sizes (e.g., the MPO and PPO) (Kadlec et al., 2023). By preserving valuable information from multiple effects within each study, the three-level meta-analysis enhances statistical power and provides a more accurate representation of effect sizes (Assink and Wibbelink, 2016). This approach decomposes variance into sampling variance (Level 1), within-study variance (Level 2), and between-study variance (Level 3), accounting for correlated and hierarchical effects (Cheung, 2019). For the three-level model, parameters were estimated using the REML, and results were cross-verified using the maximum likelihood (ML) method to ensure stability.

Tests of individual coefficients and their corresponding 95% confidence intervals (95% CIs), were based on a t-distribution (Jukic et al., 2023). Additionally, prediction interval (PI) were calculated based on the t-distribution, which provides useful additional information compared to the 95% CI, especially considering the use of a random-effects model (Borg et al., 2024). Heterogeneity was assessed using *I²* and PI, with *I²* categorized as *low* (0–25%), *moderate* (25–50%), *substantial* (51–75%), or *considerable* (76–100%) (Higgins and Thompson, 2002). The statistical significance threshold was set at *p* < 0.05.

To explore sources of heterogeneity among studies and assess potentially moderating factors, this meta-analysis employed subgroup analysis and meta-regression analysis, conducting statistical analyses on continuous variables (Hopkins and Batterham, 2018). Moderator analyses were conducted when there were at least 3 studies (Cumpston et al., 2019) and at least 10 studies available for each meta-regression (Ruppar, 2020) to explore potential sources of heterogeneity. The following variables were included in the subgroup analysis: (a) sex group; (b) training status; (c) CAF dose; (d) CAF form; (e) interval type.

Based on previous participant categorization frameworks, training status was classified as recreationally active, trained, and well-trained (McKay et al., 2022). HIIE interval time were categorized as follows based on Buchheit and Laursen (Buchheit and Laursen, 2013) and Girard (Girard et al., 2011): short intervals (duration < 60s per repetition), intermittent-sprint exercise (ISE, repeated sprints with duration < 10s for each sprint, recovery > 60s), RSA (repeated sprints with duration < 10s for each sprint, recovery < 60s).

We conducted a mix-effects meta-regression analysis with REML estimation, recognized for its robustness (Zuur et al., 2009). To assess the shape of relationships, we fitted linear and quadratic functions and compared the models, selecting the model with the lowest bias-corrected Akaike information criterion (Harrell, 2015). All analyses were conducted using the ‘metafor’ package, and results were visualized using ‘ggplot2’ (Gustavsson et al., 2022). Statistical power was calculated for each subgroup and the overall pooled effect to assess the risk of false negatives. These calculations were performed using the ‘metameta’ package (Quintana, 2023).

#### 2.6.3 Publication bias and sensitivity analysis

Funnel plots (Peters et al., 2008), Egger’s asymmetry test (Egger et al., 1997), and trim-and-fill tests were used to assess publication bias (with analyses conducted only when k > 10 (Sterne et al., 2011)). A *p*-value > 0.05 was considered indicative of no publication bias.

We conducted sensitivity analyses using a leave-one-out method to assess the robustness of the primary pooled effects in two-level and three-level meta-analyses. For three-level meta-analysis, Cook’s distance (Viechtbauer and Cheung, 2010) and studentized residuals (Atkinson et al., 1983) were used at both the within-study level (level 2) and the between-study level (level 3). Observations were flagged as potentially influential if their hat values or Cook’s distances exceeded three times their respective means, or if the absolute value of studentized residuals exceeded three. The three-level model was then re-estimated with outliers excluded to examine the robustness of the findings.

### 2.7 Certainty of the evidence

The risk of bias was considered in the interpretation of the results by applying the Grading of Recommendations Assessment, Development, and Evaluation (GRADE) methodology. Evidence was rated as “high” (further research is very unlikely to change our confidence in the estimate of effect), “moderate” (further research is likely to have an important impact on our confidence in the estimate of effect and may change the estimate), “low” (further research is very likely to have an important impact on our confidence in the estimate of effect and is likely to change the estimate) or “very low” (any estimate of effect is very uncertain) (Schünemann et al., 2019). GRADE assessment was evaluated by one reviewer (P.Y.) and independently reviewed by a second reviewer (X.K.).

## 3 Results

### 3.1 Studies retrieved

**Figure 1:**
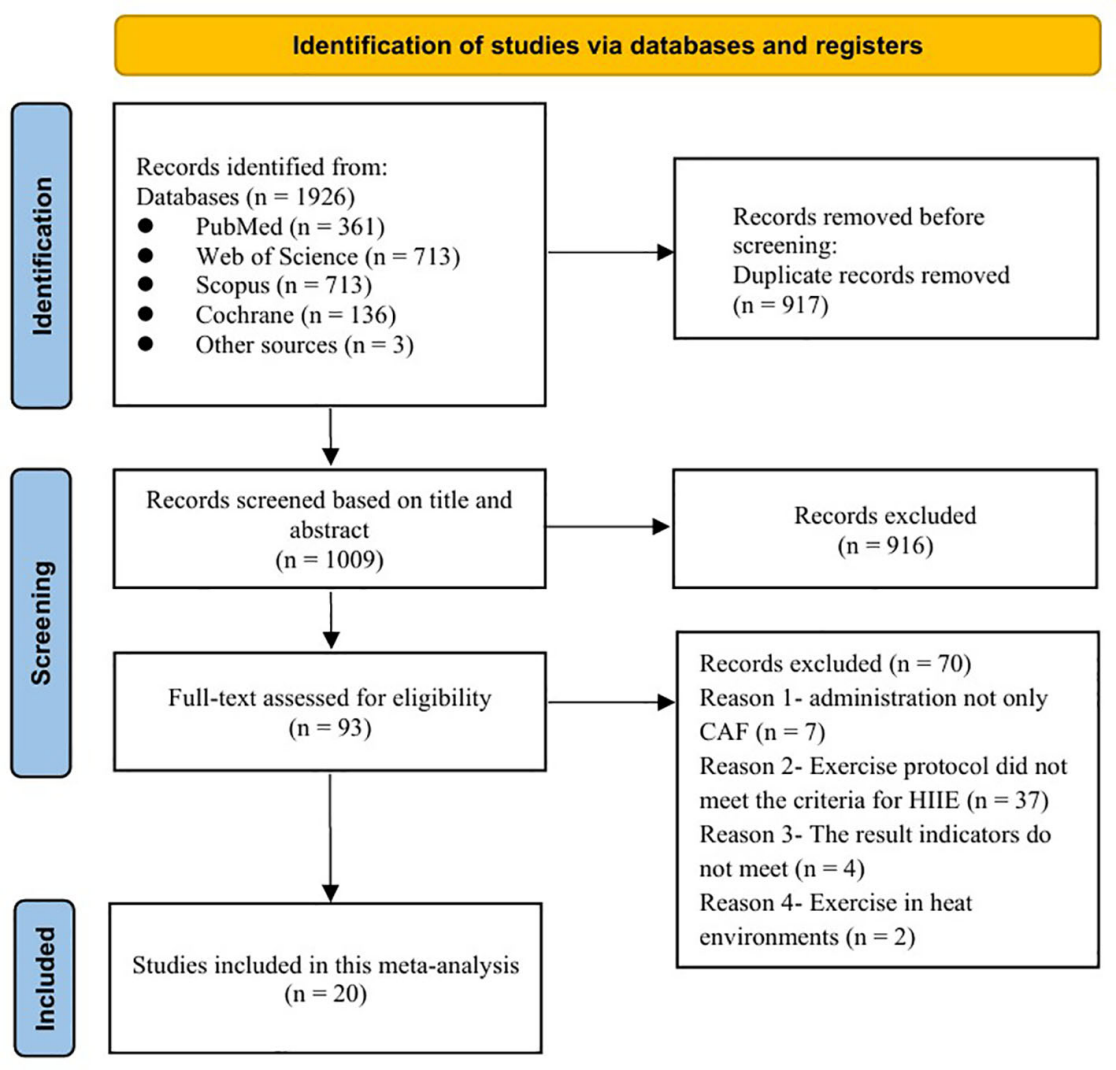
Flow diagram of study selection.

The initial search yielded 1926 publications, comprising 1923 records identified through the primary database search and an additional 3 from google research. After screening and assessment of eligibility, 20 studies were included in the meta-analysis (**Figure 1**).

### 3.2 Characteristics of included studies

**Table 1** shows the main characteristics of all included studies. The total participant pool comprised 320 individuals, including 263 males and 57 females. Individual study sample sizes ranged from 8 to 52 participants. CAF withdrawal periods ranged from 8 to 72 h. The HIIE recovery time ranged from 10s to 300s and sprint durations from 4s to 60s. CAF forms included capsules, beverages, and gum. Participant training status included recreational active, trained, and well-trained athletes.

**Table 1:** Study characteristics.

Study	Sample + age (years) + body mass + training status	Habitual caffeine intake (mg/day) + caffeine withdrawal (h)	Caffeine dose (mg/kg)		Timing (min)	Caffeine form	HIIE protocol	Rest/work ratio	Outcomes
Paton et al., 2001	16 M; 22 ± 3; 79 ± 9; recreational active	N.A.; 48	6	60		capsules	10 × 10s running (maximal-effort sprints) with 10s active recovery	1	⑥
Crowe et al., 2006	12 M, 5 F; 21.1 ± 3.0; 73.3 ± 10.5; recreational active	80–200; 24	6	72		beverage	2 × 60s cycling (maximal-effort sprints) with 180s recovery	3	②③❶❷❸❹
Schneiker et al., 2006	10 M; 20 ± 3; 77.7 ± 13.9; recreational active	N.A.; 48	6	60		capsules	2 × (18 × 4s cycling [maximal-effort sprints] with 100s [35% VO _2peak_]+ 20s recovery)	30	②③❷
Carr et al., 2008	10 M; 25 ± 5; 85.25 ± 13.98; trained	N.A.; 48	6	60		capsules	3 × (6 × 20 m running [maximal-effort sprints] with 25s recovery) + 2 × (6 × 20 m running [maximal-effort sprints] with 60s recovery)	5.4	④⑤❷
Glaister et al., 2008	21 M; 21 ± 3; 77.7 ± 13.5; recreational active	88.4 ± 87.3; 48	5	60		capsules	12 × 30 m running (maximal-effort sprints) with 35s active recovery	7.8	⑤⑥❶❷❸
Paton et al., 2010	9 M; 24 ± 7; 62.5 ± 5.4; well-trained	< 300; 72	240 mg	5		gum	2 × (10 × 30s cycling [maximal-effort sprints] with 30s recovery)	1	①
Lee et al., 2012a	14 M; 18.7 ± 0.8; 67.7 ± 6.2; recreational active	> 200; 72	6	60		capsules	2 × (12 × 4s cycling [maximal-effort sprints] with 20s recovery)	5	①②③❷❸
Lee et al., 2012b	14 M; 18.7 ± 0.8; 67.7 ± 6.2; recreational active	> 200; 72	6	60		capsules	2 × (12 × 4s cycling [maximal-effort sprints] with 90s recovery)	22.5	①②③❷❸
Del Coso et al., 2012	19 M; 21 ± 2; 67 ± 2; trained	< 60; 48	3	60		beverage	7 × 30 m running (maximal-effort sprints) with 30s active recovery	6.7	⑦
Lee et al., 2014a	12 M; 20.4 ± 1.1; 75 ± 9; recreational active	N.A.; 72	6	70		capsules	10 × (5 × 4s cycling [maximal-effort sprints] with 20s recovery)	5	①②③❶❷❸❹
Lee et al., 2014b	8 F; 21.3 ± 1.2; 58.6 ± 7.3; well-trained	50–100; 48	6	60		capsules	10 × (5 × 4s cycling [maximal-effort sprints] with 20s recovery)	5	①②③❸
Study	Sample + Age (years) + Body mass + Training status	Habitual caffeine intake (mg/day) + Caffeine withdrawal (h)	Caffeine dose (mg/kg)	Timing (min)		Caffeine form	HIIE protocol	Rest/work ratio	Outcomes
Lara et al., 2014	18 F; 21 ± 2; 57.8 ± 7.7; trained	N.A.; 48	3	60		beverage	7 × 30 m running (maximal-effort sprints) with 30s active recovery	6.7	⑦
Evans et al., 2018	18 M; 21.2 ± 1.1; 80.4 ± 6.6; recreational active	N.A.; 24	200 mg	5		gum	10 × 40 m running [maximal-effort sprints] with 30s active recovery	3.4	⑤⑥❷
Fowles et al., 2021	13 M; 32 ± 11; 65.7 ± 5.9; trained	190 ± 134; 12	2.08	60		beverage	4 × 60s cycling (maximal-effort sprints) with 5 min active recovery	5	①②③❶❸
Kopec et al., 2016	11 M; 20 ± 2; 74.5 ± 8.2; trained	< 160; 24	6	60		capsules	3 × (6 × 20 m running maximal-effort sprints with 25s recovery) + 2 × (30 × 1min [1 × agility + 1 × sprints + 2 × jogging + 3 × walking])	7.1	④⑤❶❷
Dos Santos Quaresma et al., 2021	10 M; 26.9 ± 4.0; non-athlete	200–400; 8	6	60		beverage	60s cycling (90% W _max_) with 120s (50% W _max_) recovery	2	❸❹
Bezerra et al., 2022	10 M; 3.6 ± 3.3; 71.2 ± 8.7 recreational active	N.A.; 24	6	60		capsules	6 × IAR (maximal-effort sprints) with 60s active recovery	3.4	④⑤⑥❶❸
Salgueiro et al., 2022	10 M; 18.2 ± 1.7; 72.7 ± 10.8; trained	N.A.; 48	6	60		capsules	10 × 400 m swimming (maximal-effort sprints) with 60s active recovery	0.2	⑦❷❸
Bernardo et al., 2024	12 M; 26 ± 4; 80.7 ± 7.6 recreational active	198 ± 114; 24	6	60		capsules	12 × 6s cycling (maximal-effort) with 60s active recovery	10	②③❷❸❹
Pérez-López et al., 2025 ^a^	26 M; 24.6 ± 4.5; 76.3 ± 10.7; trained	68 ± 61; 72	3	60		beverage	4 × 30s cycling (maximal-effort sprints) with 90s recovery	3	①②❷
Pérez-López et al., 2025 ^b^	26 F; 24.6 ± 4.5; 76.3 ± 10.7; trained	68 ± 61; 72	3	60		beverage	4 × 30s cycling (maximal-effort sprints) with 90s recovery	3	①②❷
Matsumura et al., 2025	16 M; 20 ± 1; 66.0 ± 4.3; well-trained	226 ± 168; 72	6	60		capsules	3 × 30s cycling (maximal-effort sprints) with 120s recovery	4	①②

a, b represents difference trials. M, male. F, female. W _max_, maximal power output. CAF, caffeine; PLA, placebo; HIIE, high-intense interval exercise; IAR, Illinois agility run; N.A., represents that the value is not available. The symbols ①, ②, ③, ④, ⑤, ⑥, ⑦, ❶, ❷, ❸, and ❹ represent mean power output, peak power output, total work, total time, fastest time, average time, speed, heart rate, blood lactate concentration, ratings of perceived exertion, and glucose.

### 3.3 Primary analysis

**Figure 2:**
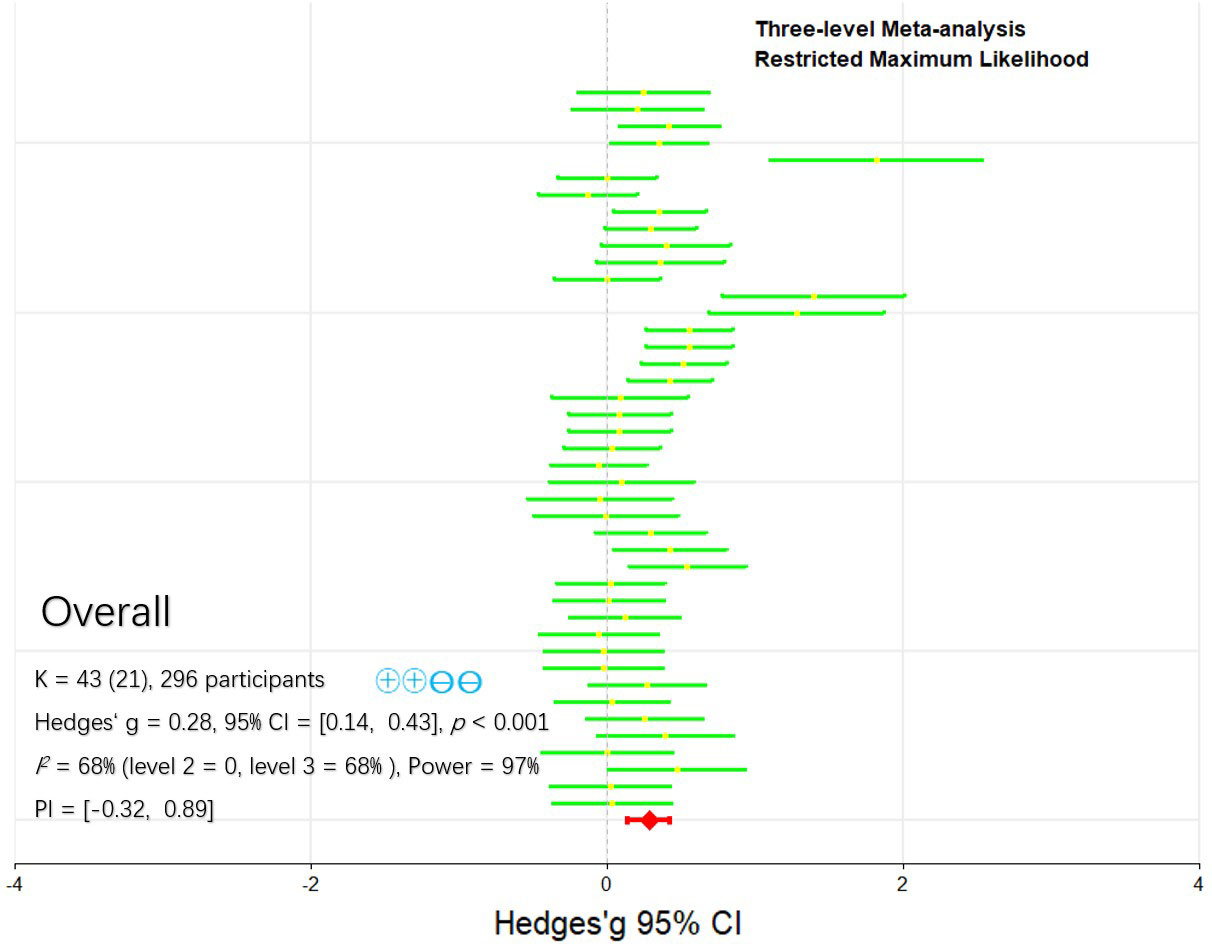
Primary pooled effect sizes for acute caffeine supplementation on overall HIIE performance. K, the total number of effects included in the pooled effect size; Hedge’s *g*, the effect size indicators used in the pooled; 95% CI, 95% confidence interval; PI, prediction interval; *p* value, statistically significant *p* values for pooled results; *I^2^*, quantitative indicators of heterogeneity; Power, statistical power for pooled effect size; Blue circles, Grade, grading of recommendations assessment, development and evaluation, a system for evaluating the quality of evidence and strength of recommendations.

For overall HIIE performance, the three-level meta-analysis showed that CAF ingestion significantly improved HIIE performance (*g* = 0.28, 95% CI: 0.14 to 0.43 *p* < 0.001) (**Figure 2**), In addition, variance decomposition showed that there was no within-study variance (Level 2, 0%). The overall heterogeneity was driven entirely by variance between studies (Level 3, 68%), of which 32% was attributable to sampling error (Level 1). In accordance with the recommendations of Hunter and Schmidt (Raudenbush, 1991), significant heterogeneity is considered present when the total variance explained by sampling error is less than 75%. Consequently, we proceeded with a moderator analysis to investigate the sources of this heterogeneity.

**Figure 3:**
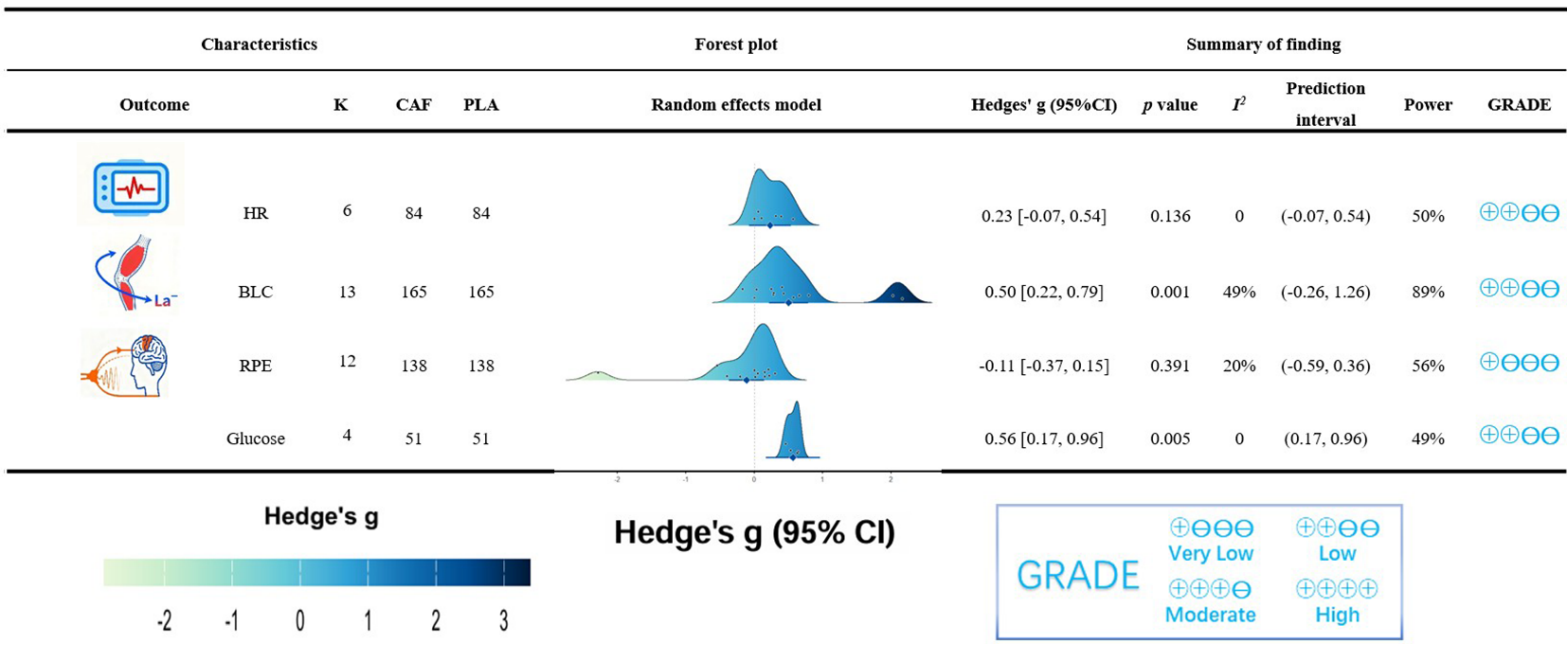
Effect of caffeine ingestion in HIIE as compared to placebo on HR, BLC, glucose and RPE. 95% CI, 95% confidence interval; Hedge’s *g*, the effect size indicators used in the pooled; *I^2^*, quantitative indicators of heterogeneity; K, the total number of included studies; CAF, caffeine-supplement group; PLA, placebo-supplement group; *p* value, statistically significant *p* values for pooled results; HR, heart rate; BLC, blood lactate concentration; RPE, ratings of perceived exertion.

Regarding physiological responses, the two-level meta-analysis showed that CAF ingestion significantly increased glucose (*g* = 0.56, 95% CI: 0.17 to 0.96, *p* = 0.005) following CAF ingestion during HIIE. Additionally, a significant elevation in BLC was observed (*g* = 0.50, 95% CI: 0.22 to 0.79, *p* < 0.001). However, there was no significant difference in HR (*g* = 0.23, 95% CI: -0.07 to 0.54, *p* = 0.126) between CAF and placebo conditions (**Figure 3**).

Regarding perceptual responses, the meta-analysis found no significant difference in RPE (*g* = -0.11, 95% CI: -0.37 to 0.15, *p* = 0.391) between CAF and placebo during HIIE (**Figure 3**).

### 3.4 Moderator analysis

We conducted moderator analysis to explore the modifying effects of sex, training status, CAF form, CAF dose, interval type, and recovery time on overall HIIE performance.

#### 3.4.1 Potential moderators of participant characteristics

**Figure 4:**
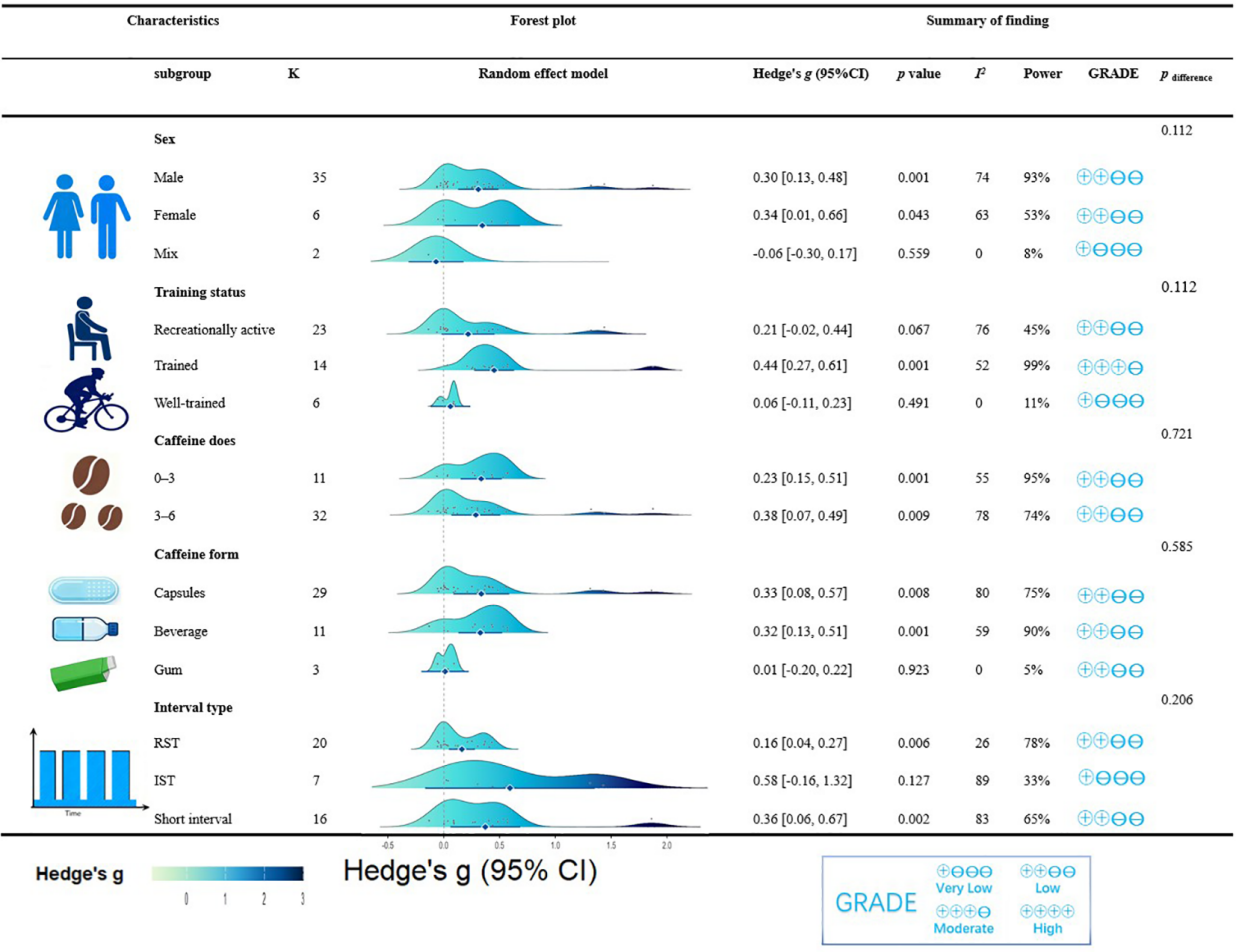
Subgroup analysis by sex, training status, caffeine form, interval type, recovery time on the effect of the caffeine ingestion on overall HIIE performance. 95% CI, 95% confidence interval; Hedge’s *g*, the effect size indicators used in the pooled; *I^2^*, quantitative indicators of heterogeneity; K, the total number of effects included in the pooled effect size; CAF, caffeine-supplement group; PLA, placebo-supplement group; *p* value, statistically significant *p* values for pooled results; *p*_difference_, *p* value of the difference between subgroups; RSA, repeated sprint ability; ISE, intermittent-sprint exercise.

Moderator analyses indicated that CAF significantly improved in male group (*g* = 0.30, 95% CI: 0.13 to 0.48, *p* < 0.001) and female group (*g* = 0.34, 95% CI: 0.01 to 0.66, *p* = 0.043) during HIIE, but not in mix group (*g* = -0.06, 95% CI: -0.30 to 0.17, *p* = 0.599). Furthermore, CAF significantly improved HIIE performance in trained athlete (*g* = 0.44, 95% CI: 0.27 to 0.61, *p* < 0.001), but no significant effect on recreationally active (g = 0.21, 95% CI: -0.02 to 0.44, *p* = 0.067) and well-trained (*g* = 0.06, 95% CI: -0.11 to 0.23, *p* = 0.491). Additionally, there was no significant difference in overall HIIE performance between different subgroups in terms of sex, training status (all *p* for subgroup > 0.05) (**Figure 4**).

#### 3.4.2 Potential moderators of supplementation protocol

Moderator analyses demonstrated that CAF ingestion significantly improved overall HIIE performance in capsule form (*g* = 0.33, 95% CI: 0.08 to 0.57, *p* = 0.008) and in beverage form (*g* = 0.32, 95% CI: 0.13 to 0.51, *p* < 0.001), but not in gum form (*g* = 0.01, 95% CI: -0.20 to 0.22, *p* = 0.923). Furthermore, low dose (*g* = 0.33, 95% CI: 0.15 to 0.51, *p* < 0.001) and moderate dose (*g* = 0.28, 95% CI: 0.07 to 0.49, *p* = 0.009) of CAF significantly enhanced HIIE performance, but there was no significant difference in overall HIIE performance between different subgroups in terms of CAF dose and CAF form (all *p* for subgroup > 0.05) (**Figure 4**).

#### 3.4.3 Potential moderators of HIIE protocol

Moderator analyses demonstrated that CAF ingestion significantly improved RSA (*g* = 0.16, 95% CI: 0.04 to 0.27, *p* = 0.006) and short intervals (*g* = 0.36, 95% CI: 0.06 to 0.67, *p* = 0.002), but not in ISE (*g* = 0.58, 95% CI: -0.16 to 1.32, *p* = 0.127). Additionally, no significant difference was observed between interval type (*p* for subgroup > 0.05) (**Figure 4**).

**Figure 5:**
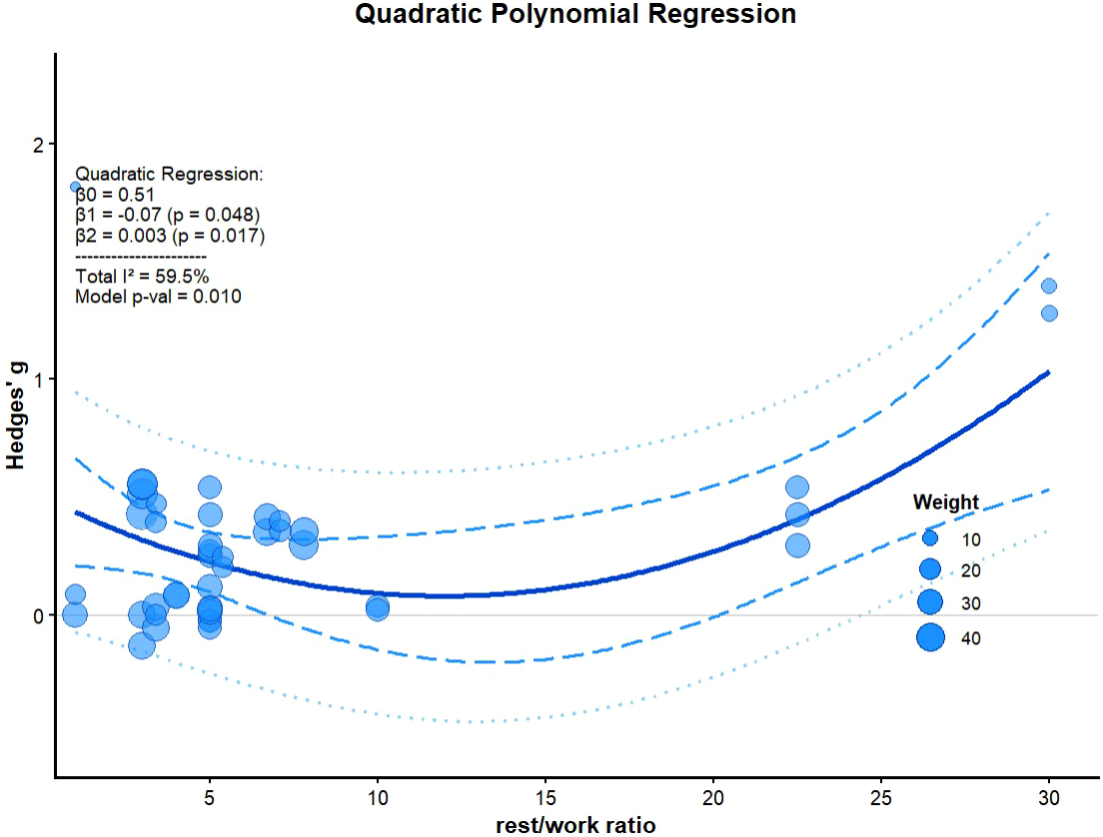
Regression analysis of rest/work ratio. *β_0_*, the regression intercept; *β_1_* and *β_2_*, the regression slopes; *I^2^*, heterogeneity; the blue shaded part represents the 95% confidence interval; the outermost blue dotted line represents the prediction interval; *p* value, statistically significant *p* values for regression analysis results.

Regarding the rest/work ratio of HIIE, a significant linear association was detected (*β_1_* = -0.07, *p* = 0.048) and further examination of nonlinear relationships using polynomial meta-regression also revealed significant effects (*β_2_* = 0.003, *I^2^* = 60%, *p* = 0.017) (**Figure 5**).

### 3.5 Risk of bias and quality of methods

The risk of bias assessment was conducted for the 20 included studies (Paton et al., 2001, 2010; Crowe et al., 2006; Schneiker et al., 2006; Carr et al., 2008; Glaister et al., 2008; Del Coso et al., 2012; Lee et al., 2012, 2014b, 2014a; Lara et al., 2014; Kopec et al., 2016; Evans et al., 2018; Dos Santos Quaresma et al., 2021; Fowles et al., 2021; Bezerra et al., 2022; Salgueiro et al., 2022; Bernardo et al., 2024; Matsumura et al., 2025; Pérez‐López et al., 2025) (**Supplementary Appendix S6**). Fourteen trials had a *low* risk of bias, while six were deemed to have a moderate risk of bias.

Regarding PEDro scores, the 20 included studies had scores ranging from 6 to 10 (**Supplementary Appendix S2**). The mean rating was 8.6, indicating that the overall collection of studies was of good quality. Out of these, four studies were rated as excellent, fifteen studies were rated as good, and one study was rated as fair. Notably, no study reported the blinding of all assessors (item 7).

The risk of publication bias was assessed using a funnel plot combined with Egger’s test and trim-and-fill tests. Egger’s regression suggested potential publication bias for the pooled effects on overall HIIE performance and BLC (*p* < 0.05). However, trim-and-fill analysis showed that the pooled effects remained significant (**Supplementary Appendix S5**), suggesting that the main findings were not substantially affected by publication bias.

### 3.6 Sensitivity analysis

#### 3.6.1 Sensitivity analysis for primary effect

**Figure 6:**
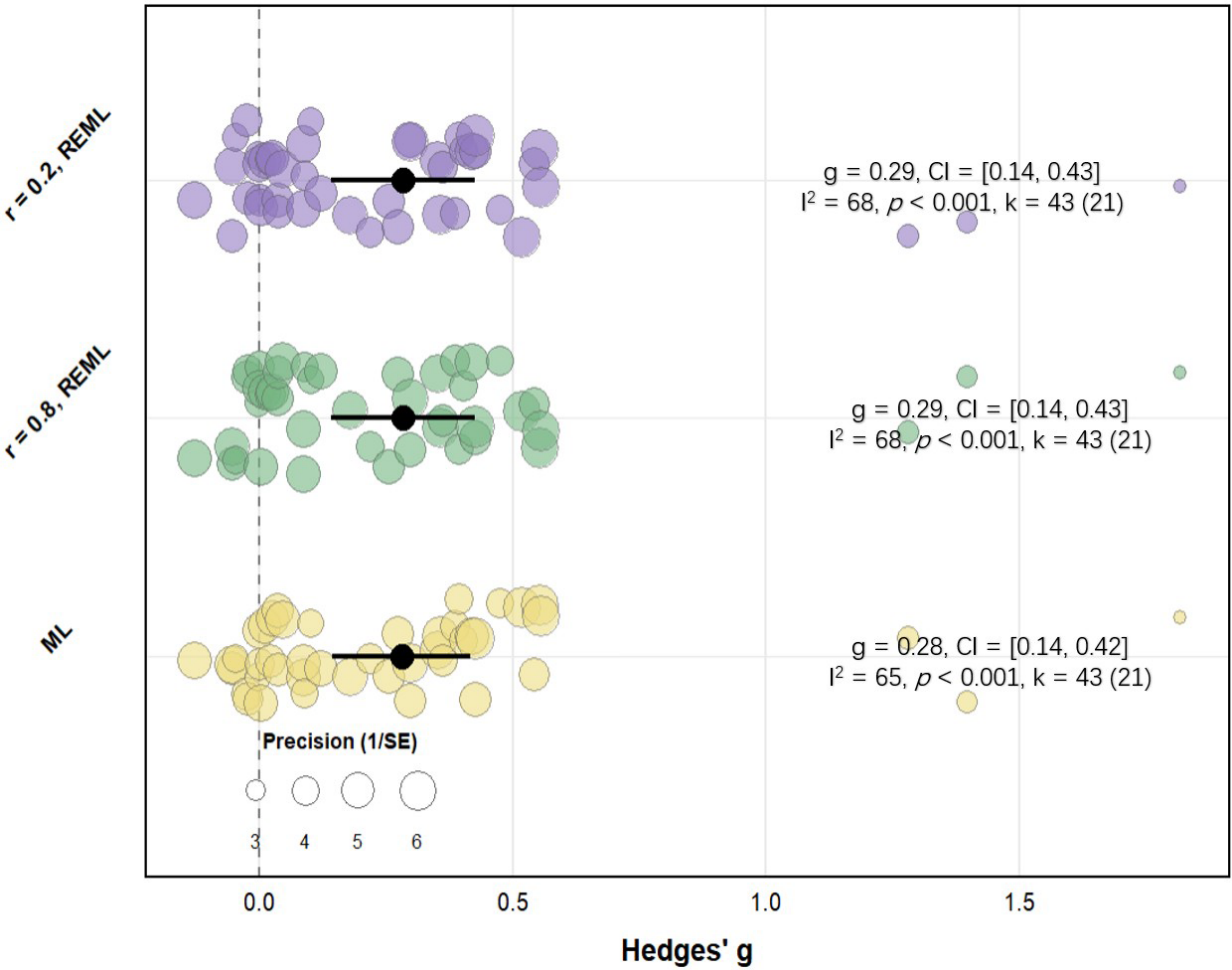
Sensitivity analysis of primary effects. K, the total number of effects included in the pooled effect size; Hedge’s *g*, the effect size indica tors used in the pooled; CI, 95% confidence interval; *p*-value, statistically significant *p* values for pooled results; r, correlation coefficient between caffeine group and PLA group; ML, maximum likelihood model; *I^2^*, quantitative indicators of heterogeneity.

To assess the robustness of the main effect, we conducted a sensitivity analysis using two distinct values of the within-subject correlation coefficient (r) for crossover studies: a relatively low value (r = 0.2) and a relatively high value (r = 0.8). The results revealed no meaningful differences in the pooled effect size; all corresponding *p*-values remained statistically significant (*p* < 0.001) (**Figure 6**). The within-subject correlation coefficient had no impact on heterogeneity estimates, further indicating that different correlation assumptions exerted limited influence on heterogeneity estimation. Results from the ML and REML models were comparable.

Leave-one-out sensitivity analyses were conducted for both the three-level and two-level meta-analyses. The results indicated that the findings were robust across both models (**Supplementary Appendix S4**).

In three-level meta-analysis, Schneiker et al. (2006) and Salgueiro et al. (2022) were flagged as outliers based on Cook’s distance and studentized residuals. Excluding these studies did not significantly alter the re-pooled results (k = 40, *g* = 0.20, 95% CI = 0.11 to 0.30, *I^2^* = 39% [*moderate*], *p* < 0.01, PI [−0.12, 0.53], statistical power = 99%). After outlier exclusion, heterogeneity decreased, and re-pooled estimates were numerically more stable. Regarding BLC, Schneiker et al. (2006) and Carr et al. (2008) were identified as outliers based on both Cook’s distance and studentized residuals, yet removing those studies did not substantially change the re-pooled results (k = 11, *g* = 0.35, *p* < 0.001). For RPE, Salgueiro et al. (2022) was flagged as an outlier based on Cook’s distance and studentized residuals, but the re-pooled results remained statistically unchanged after removal (k = 11, *g* = -0.01, *p* = 0.942).

#### 3.6.2 Sensitivity analysis for moderator effect

Sensitivity analyses involving the exclusion of influential cases revealed no substantial alterations in the estimates of several moderators within the three-level meta-analytic model.

Sensitivity analyses were conducted using a leave-one-out method for all moderator analyses. Our findings showed that the exclusion of certain studies resulted in notable changes to the pooled results. For female, removing Lara et al. (2014) and Lee et al. (2014a) changed the pooled results from significant to non-significant. For recreationally active, excluding Schneiker et al. (2006) altered the pooled results from non-significant to significant (**Supplementary Appendix S4**).

## 4 Discussion

The main findings indicate that CAF administration significantly enhances HIIE performance. However, the magnitude and consistency of this effect appear to be influenced by several moderating factors, including training status, CAF form, and interval type. Additionally, variations in the rest/work ratio were also found to play a critical role in modulating the observed outcomes.

### 4.1 Effect of CAF on HIIE

Our results indicate that CAF ingestion significantly enhances overall HIIE performance (*g* = 0.28, 95% CI = 0.14 to 0.43) (**Figure 2**). This finding aligns with a previous meta-analysis showing that 3–6 mg/kg of CAF significantly improves HIIE-related performance (*g* = 0.14, 95% CI = 0.03 to 0.25) (Salinero et al., 2019), and the effect appears to be more pronounced during high-intensity competition (Diaz-Lara et al., 2024). This discrepancy may be attributed to complex contextual confounders (e.g., motivation, decision-making, game dynamics), ultimately leading to an overestimation of CAF’s actual efficacy. Mechanistically, performance decrements during HIIE are primarily driven by central fatigue (e.g., adenosine accumulation) and peripheral mechanisms (e.g., phosphocreatine depletion) (Nehlig et al., 1992; Gaitanos et al., 1993). CAF acts as an adenosine receptor antagonist, modulating both central and peripheral physiological pathways (Guest et al., 2021).

Centrally, this antagonism increases cortical excitability and attenuates central fatigue (Davis et al., 2003; Cristina-Souza et al., 2022). Consistent with the higher exercise outputs observed after CAF ingestion, our meta-analysis also showed increased BLC (**Figure 3**). This is likely driven by greater sympathetic activation (Porto et al., 2022). Accordingly, higher BLC accumulation reflects increased glycolytic flux (Gastin, 2001; Buchheit and Laursen, 2013). Thus, an enhanced ability to generate lactate during HIIE suggests a greater capacity for anaerobic energy provision, thereby enabling the maintenance of higher exercise intensities (Bishop, 2010).

Reduction in RPE is typically a highly correlated outcome associated with CAF’s ergogenic effects (Grgic et al., 2019). Our meta-analysis revealed no significant differences in RPE between CAF and placebo conditions (**Figure 3**), despite clear improvements in HIIE performance. Collectively, these observations suggest that CAF may increase central tolerance to fatigue-related afferent signals (Davis et al., 2003; Cristina-Souza et al., 2022), enabling athletes to sustain higher work outputs without a corresponding increase in RPE.

Peripherally, given that high-intensity exercise heavily relies on blood glucose (Wahren et al., 1971; Ahlborg et al., 1974; Coggan et al., 1995; Wasserman, 2009), CAF facilitates muscle-glucose uptake by stimulating calcium release from the sarcoplasmic reticulum and increasing calmodulin activity (Youn et al., 1991; Terada et al., 2003). This accelerated uptake expedites muscle glycogen depletion, triggering the upregulation of IL-6 (Pedersen and Febbraio, 2008, 2012). Elevated IL-6 can promote hepatic glucose output by stimulating gluconeogenesis and glycogenolysis (Pedersen et al., 2004), ultimately driving the observed elevation in glucose (**Figure 3**).

### 4.2 Potential moderators of HIIE protocol

Meta-regression revealed that the rest/work ratio (*β_2_* = 0.003, *p* = 0.017) moderated the effect of CAF on HIIE in a U-shaped manner (**Figure 5**), indicating that both lower and higher ratios yield greater performance enhancements. Although between-subgroup differences for interval types did not reach statistical significance (*p* > 0.05), the pattern of effect sizes was consistent with the U-shaped relationship demonstrated in the regression analysis.

Short intervals (rest/work ratio 0.2–5) predominantly occupy the left arm of the U-shaped regression. Subgroup analysis showed that CAF significantly enhanced performance in short intervals (*g* = 0.36, 95% CI: 0.06 to 0.67) (**Figure 4**). Short intervals typically involve longer sprint durations (Spencer et al., 2005), thereby eliciting a higher relative contribution from aerobic metabolism and inducing substantial central and peripheral fatigue (Buchheit and Laursen, 2013). CAF, as an adenosine receptor antagonist, activates the central nervous system to counteract this fatigue. In these high-density scenarios, CAF appears highly effective in mitigating fatigue accumulation.

RSA (rest/work ratio 1–7.8) are primarily distributed in the mid-left section of the U-shaped regression curve. Subgroup analysis showed that CAF elicited a small but significant improvement in RSA (*g* = 0.16, 95% CI: 0.04 to 0.27) (**Figure 4**). However, a previous meta-analysis by Lopes-Silva et al. (2019) reported that CAF ingestion had no significant effect on RSA, which may be attributed to their omission of three relevant studies and a less comprehensive selection of performance metrics. Mechanistically, RSA typically involves high-intensity sprints (< 10s) interspersed with short recovery periods (< 60s) (Spencer et al., 2005), where ATP resynthesis heavily relies on phosphocreatine (PCr) (~50%) and anaerobic glycolysis (~40%) (Gaitanos et al., 1993). Consequently, the rapid accumulation of anaerobic metabolites may attenuate the ergogenic efficacy of CAF (Bishop, 2010).

ISE (rest/work ratio 10–30) are primarily distributed on the right side of the U-shaped regression curve. Although a moderate effect size was observed (*g* = 0.58, 95% CI: -0.16 to 1.32) (**Figure 4**), it did not reach statistical significance, potentially due to low statistical power (33%) in this subgroup, which could lead to a false negative. Compared with RSA, ISE typically involves extended recovery periods (60–300s), allowing for near-complete metabolic recovery (Balsom et al., 1992; Duffield et al., 2009). The minimization of metabolic fatigue likely allows CAF’s ergogenic effects to manifest through non-metabolic mechanisms, such as direct local adenosine receptor antagonism (Cristina-Souza et al., 2022), which enhances excitation–contraction coupling and increases Na^+^-K^+^ pump activity (Lindinger et al., 1993), thereby maintaining muscle excitability. The distinct ergogenic mechanisms of CAF under different metabolic conditions may also help explain the U-shaped relationship demonstrated in our meta-regression.

In conclusion, due to substantial heterogeneity and the scarce data available at higher rest/work ratios (with most studies concentrated on the left), the significant upward trend observed must be viewed conservatively. Furthermore, it is crucial to acknowledge that results drawn from meta-regression are observational, not causal. Confirming these findings requires randomized controlled trials with adequate statistical power.

### 4.3 Potential moderators of participant characteristics

#### 4.3.1 Sex

Our analysis observed similar trends in enhancing HIIE performance among male (*g* = 0.30, 95% CI = 0.13 to 0.48) and female participants (*g* = 0.34, 95% CI = 0.01 to 0.66), with no significant between-group differences (*p* for subgroup > 0.05) (**Figure 4**). These findings indicate that the overall ergogenic effects of CAF on HIIE are not strictly sex-dependent. This aligns with mechanistic evidence demonstrating that the general pharmacokinetic parameters of CAF (including absorption, distribution, metabolism, and excretion) are largely comparable between adult males and females (Magkos and Kavouras, 2005). Although CYP1A2-mediated caffeine clearance in women may be influenced by hormonal fluctuations and oral contraceptive use (Kamimori et al., 1999; Wikoff et al., 2017), such pharmacokinetic differences do not appear to consistently translate into meaningful sex-based differences in acute performance responses, particularly at doses below 6 mg/kg (Van Soeren and Graham, 1998).

#### 4.3.2 Training status

Although the effects across training-status groups appeared inconsistent, training status did not significantly moderate the effects of CAF (*p* for subgroup > 0.05) (**Figure 5**). Pooled estimates suggested the favorable effects on trained athletes (*g* = 0.44, 95% CI = 0.27 to 0.61), whereas effects on recreationally active (*g* = 0.21, 95% CI = -0.02 to 0.44) and well-trained athletes (*g* = 0.06, 95% CI = -0.11 to 0.23) (**Figure 4**) were not statistically significant. Compared with recreationally active individuals, trained athletes may be better able to sustain the high intensity and duration required to fully capitalize on the stimulatory effects of CAF (Graham, 2001; Guest et al., 2021; Khodadadi et al., 2025). In contrast, the smaller estimate observed in well-trained athletes may reflect a reduced scope for further improvement because of near-maximal physiological adaptations (Maughan et al., 2018; Southward et al., 2018). Importantly, pooled estimate in recreationally active athletes shifted from negative to positive after sensitivity analysis (**Supplementary Appendix S4**), suggesting that this finding should be interpreted with caution.

### 4.4 Potential moderators of supplementation protocols

#### 4.4.1 CAF form

Although no statistically significant differences were observed among the three delivery forms (*p* for subgroup > 0.05) (**Figure 4**), our moderator analyses suggested that the pooled effect estimates were positive for CAF administered as capsules (*g* = 0.33, 95% CI = 0.08 to 0.57) and beverages (*g* = 0.32, 95% CI = 0.13 to 0.51), but not as gum (*g* = 0.01, 95% CI = -0.20 to 0.22) (**Figure 4**). This aligns with a network meta-analysis by Xue et al. (2025), showing that moderate CAF doses (4–6 mg/kg) significantly improved time-trial performance via capsules (*g* = -0.31, 95% CI = -0.54 to -0.17), but not in gum (*g* = 0.00, 95% CI = -0.54 to 0.53). The lack of a consistent effect for CAF gum may be attributed to its limited and variable systemic absorption. Specifically, residual CAF in discarded gum reduces the actual administered dose (Kamimori et al., 2002; Whalley et al., 2021), resulting in lower, less predictable circulating concentrations. Conversely, capsules and beverages ensure precise dosing, yielding reliable serum CAF peaks that maximize performance enhancement. Furthermore, psychological expectancies may further augment these physiological responses, the familiarity of capsules and the sensory feedback of beverages can both elicit potent placebo effects (Marticorena et al., 2021), potentially bolstering the observed physiological gains.

#### 4.4.2 CAF dose

Our findings show that moderate dose (4–6 mg/kg) (*g* = 0.28, 95% CI: 0.07 to 0.49) and low dose CAF (0–3 mg/kg) (*g* = 0.33, 95% CI: 0.15 to 0.51) are similarly effective in improving HIIE performance (**Figure 4**). This aligns with a previous meta-analysis showing that moderate dose (*g* = 0.30, 95% CI = 0.12 to 0.48) and low dose CAF (*g* = 0.38, 95% CI = 0.09 to 0.67) are similarly effective in improving endurance performance. Furthermore, previous study showed that the lower incidence of adverse effects (e.g., gastrointestinal discomfort and irregular heart rate) associated with low dose CAF (Spriet, 2014), this dosage may be preferable when determining the optimal dosing strategy. Individual variability should also be considered (e.g., habitual CAF intake), as regular consumers may develop tolerance (Fredholm, 1982; Nikodijević et al., 1993), potentially blunting the ergogenic efficacy of lower doses (Beaumont et al., 2017; Lara et al., 2019). Notably, the vast majority of studies included in the low-dose subgroup utilized a dosage of 3 mg/kg. Altogether, preliminary evidence suggests that 3 mg/kg of CAF appear to offer the most practical balance between HIIE performance enhancement and tolerability.

### 4.5 Practical implications

CAF pre-competition supplementation provides similar ergogenic effects on exercise performance for both male and female athletes. Furthermore, CAF use is particularly recommended for trained level athletes. Regarding administration, both capsules and beverage forms appear to be effective, and preliminary evidence suggests that low dose (~3 mg/kg) may represent the optimal balance between efficacy and tolerability. Notably, CAF is highly recommended for athletes engaged in sports characterized by extreme rest/work ratios (i.e., very low or very high ratios).

### 4.6 Future directions

Based on the findings of the present systematic review and meta-analysis, several priorities for future research are warranted. First, although our meta-regression revealed that the rest/work ratio significantly moderates CAF’s ergogenic effect on HIIE in a U-shaped manner, data concerning higher ratios (i.e., the right side of the U-curve) remain scarce. Future investigations should specifically target HIIE protocols with extended recovery periods to validate the efficacy of CAF in these scenarios and delineate more precise optimal ranges for the rest/work ratio. Second, future research should adopt a more granular classification of training status (e.g., recreationally active, trained, and well-trained/elite) to better determine whether CAF’s ergogenic effects meaningfully differ across athlete levels. This refined stratification is essential to elucidate potential ceiling effects or psychological moderators unique to elite populations. Third, while our results preliminarily suggest that low dose (~3 mg/kg) may represent a pragmatic dose, subsequent studies should explicitly account for inter-individual variability. Factors such as habitual CAF intake, tolerance, and relevant genetic polymorphisms (e.g., CYP1A2) should be integrated to identify individualized dose–response relationships and optimal dosing strategies. Finally, although our analysis demonstrated the efficacy of capsules and beverages but not gum, methodological heterogeneity across studies may have influenced these results. Future trials examining different delivery forms (especially gum vs. capsules) should implement rigorous double-blind or even triple-blind designs with standardized administration protocols (e.g., controlling for absorption time and chewing duration). This will provide more definitive evidence regarding the pharmacokinetic and ergogenic differences between delivery matrices.

### 4.7 Strength and limitations

This is the first systematic review and three-level meta-analysis to comprehensively examine all performance-related outcomes from studies investigating CAF on HIIE. A comprehensive literature search was conducted across four databases to ensure the inclusion of all relevant studies. Methodologically, we employed a robust three-level meta-analytic model to account for the dependency of multiple effect sizes within studies and avoid statistical double counting. Furthermore, both the overall and moderator-level results were evaluated through sensitivity analyses to assess the stability of findings. Finally, we applied the GRADE and PEDro scale approach to evaluate the certainty of each outcome, providing a transparent assessment of evidence quality and decision-making utility.

Despite the strengths of this study, several limitations should be acknowledged. First, we were unable to examine the potential influence of genetic variability (e.g., CYP1A2/ADORA2A polymorphisms) or habitual CAF intake, as these variables were insufficiently reported in the included studies. Second, although our meta-regression identified a significant U-shaped relationship between the rest/work ratio and HIIE performance, the precise optimal range for this ratio remains undetermined. This uncertainty is primarily due to the scarcity of data points at the higher end of the rest/work spectrum (i.e., the right side of the U-curve), which precluded a more granular analysis. Third, substantial heterogeneity was present across most outcomes, likely due to variability in participant characteristics, intervention protocols, dosing strategies, and outcome assessments. Fourth, the limited number of available studies for several outcomes and subgroups reduced statistical power, thereby weakening the robustness of the estimates and limiting the generalizability of our conclusions. Finally, the sensitivity analysis (**Supplementary Appendix S4**) indicated that the results were not robust in the female and recreationally active subgroups, suggesting that these findings should be interpreted with caution.

## 5 Conclusions

This meta-analysis demonstrated that CAF significantly enhances HIIE performance. Moderator analyses indicated that these performance enhancements were present in both males and females, as well as in trained athletes. Preliminary evidence shows that CAF appeared effective when administered in capsule or beverage forms, and low dose (~3 mg/kg) may provide a practical balance of efficacy and tolerability. Meta-regression revealed the rest/work ratio moderates the effect of CAF on HIIE, indicating that both lower and higher ratios yield greater performance enhancements.

## Data Availability

The original contributions presented in the study are included in the article/**Supplementary Material**. Further inquiries can be directed to the corresponding author.
