# Supplementary material for: Does acute caffeine ingestion improve high-intensity interval exercise performance? A systematic review and meta-analysis
Source: Front Physiol. 2026 May 21;17:1858094. doi: 10.3389/fphys.2026.1858094 (PMC13233386; doi:10.3389/fphys.2026.1858094)
Supplement: Supplementary file 1 [file DataSheet1.docx]

**Supplementary Material Appendix S1 (Search strategy)**

| Data | Query | Results |
| --- | --- | --- |
| PubMed | (caffeine OR "Coffeinum N" OR Vivarin OR Durvitan OR "1,3,7-Trimethylxanthine" OR Caffedrine OR Coffeinum OR Dexitac OR coffee OR "coffee bean") AND ("High Intensity Interval Training" OR "High-Intensity Interval Training" OR "Circuit Training" OR "Interval Training" OR "High-Intensity" OR "High-Intensity Training" OR "High-Intensity Intermittent Exercise" OR "High-Intensity Intermittent Exercises" OR "High-Intensity Intermittent" OR "Sprint Interval Training" OR "high-intensity interval" OR HIT OR HIIE OR sit OR hiie OR tabata OR "Training High-Intensity Interval" OR "Exercise High-Intensity Intermittent" OR "Exercise, High-Intensity Intermittent") | 361 |
| WOS | TS=(caffeine OR "Coffeinum N" OR Vivarin OR Durvitan OR "1,3,7-Trimethylxanthine" OR Caffedrine OR Coffeinum OR Dexitac OR coffee OR "coffee bean") AND TS=("High Intensity Interval Training" OR "High-Intensity Interval Training" OR "Circuit Training" OR "Interval Training" OR "High-Intensity" OR "High-Intensity Training" OR "High-Intensity Intermittent Exercise" OR "High-Intensity Intermittent Exercises" OR "High-Intensity Intermittent" OR "Sprint Interval Training" OR "high-intensity interval" OR HIT OR HIIE OR sit OR hiie OR tabata OR "Training High-Intensity Interval" OR "Exercise High-Intensity Intermittent" OR "Exercise, High-Intensity Intermittent") | 713 |
| Scopus | TITLE-ABS-KEY((caffeine OR "Coffeinum N" OR Vivarin OR Durvitan OR "1,3,7-Trimethylxanthine" OR Caffedrine OR Coffeinum OR Dexitac OR coffee OR "coffee bean") AND ("High Intensity Interval Training" OR "High-Intensity Interval Training" OR "Circuit Training" OR "Interval Training" OR "High-Intensity" OR "High-Intensity Training" OR "High-Intensity Intermittent Exercise" OR "High-Intensity Intermittent Exercises" OR "High-Intensity Intermittent" OR "Sprint Interval Training" OR "high-intensity interval" OR HIT OR HIIE OR sit OR hiie OR tabata OR "Training High-Intensity Interval" OR "Exercise High-Intensity Intermittent" OR "Exercise, High-Intensity Intermittent")) | 713 |
| Cochrane | (caffeine OR "Coffeinum N" OR Vivarin OR Durvitan OR "1,3,7-Trimethylxanthine" OR Caffedrine OR Coffeinum OR Dexitac OR coffee OR "coffee bean"):ti,ab,kw AND ("High Intensity Interval Training" OR "High-Intensity Interval Training" OR "Circuit Training" OR "Interval Training" OR "High-Intensity" OR "High-Intensity Training" OR "High-Intensity Intermittent Exercise" OR "High-Intensity Intermittent Exercises" OR "High-Intensity Intermittent" OR "Sprint Interval Training" OR "high-intensity interval" OR HIT OR HIIE OR sit OR hiie OR tabata OR "Training High-Intensity Interval" OR "Exercise High-Intensity Intermittent" OR "Exercise, High-Intensity Intermittent"):ti,ab,kw | 136 |

| **Supplementary Material Appendix S2 (PEDro assessment)** | | | | | | | | | | | | | |  |
| --- | --- | --- | --- | --- | --- | --- | --- | --- | --- | --- | --- | --- | --- | --- |
| Study | Item 1 | Item 2 | Item 3 | Item 4 | Item 5 | Item 6 | Item 7 | Item 8 | Item 9 | Item 10 | Item 11 | Total | Rating | |
| Bernardo, M.F et al., 2024 | YES | 1 | 0 | 1 | 1 | 1 | 0 | 1 | 1 | 1 | 1 | 9 | Good | |
| Bezerra, G et al., 2022 | YES | 1 | 1 | 1 | 1 | 1 | 0 | 1 | 1 | 1 | 1 | 10 | Excellent | |
| Dos Santos Quaresma, et al., 2021 | YES | 1 | 1 | 1 | 1 | 1 | 0 | 1 | 1 | 1 | 1 | 10 | Excellent | |
| Fowles, J.R et al., 2021 | YES | 1 | 1 | 1 | 1 | 1 | 0 | 1 | 1 | 1 | 1 | 10 | Excellent | |
| Lee, C.L et al., 2014 a | NO | 1 | 1 | 1 | 1 | 1 | 0 | 1 | 1 | 0 | 1 | 8 | Good | |
| Lee, C.L et al., 2014 b | NO | 1 | 1 | 1 | 1 | 1 | 0 | 1 | 1 | 0 | 1 | 8 | Good | |
| Lee, C.L et al., 2012 | NO | 1 | 1 | 1 | 1 | 1 | 0 | 1 | 1 | 0 | 1 | 8 | Good | |
| Evans, M et al., 2018 | NO | 1 | 1 | 1 | 1 | 1 | 0 | 1 | 1 | 1 | 1 | 9 | Good | |
| Matsumura, T et al., 2025 | NO | 1 | 1 | 1 | 1 | 1 | 0 | 1 | 1 | 0 | 1 | 8 | Good | |
| Paton, C. D et al., 2010 | NO | 1 | 0 | 1 | 1 | 1 | 0 | 1 | 1 | 0 | 1 | 7 | Good | |
| Pérez-López, A et al., 2024 | NO | 1 | 1 | 1 | 1 | 1 | 0 | 1 | 1 | 0 | 1 | 8 | Good | |
| Schneiker, K. T et al., 2006 | NO | 1 | 1 | 1 | 1 | 1 | 0 | 1 | 1 | 1 | 1 | 9 | Good | |
| Paton, C. D et al., 2001 | NO | 1 | 0 | 1 | 1 | 1 | 0 | 1 | 1 | 0 | 0 | 6 | Fair | |
| Kopec, B. J et al., 2015 | NO | 1 | 1 | 1 | 1 | 1 | 0 | 1 | 1 | 1 | 1 | 9 | Good | |
| Glaister, M et al., 2008 | NO | 1 | 1 | 1 | 1 | 1 | 0 | 1 | 1 | 1 | 1 | 9 | Good | |
| Crowe, M. J et al., 2006 | NO | 1 | 1 | 1 | 1 | 1 | 0 | 1 | 1 | 1 | 1 | 9 | Good | |
| Salgueiro D et al., 2021 | NO | 1 | 0 | 1 | 1 | 1 | 0 | 1 | 1 | 1 | 1 | 8 | Good | |
| Del Coso, J et al., 2012 | NO | 1 | 0 | 1 | 1 | 1 | 0 | 1 | 1 | 1 | 0 | 7 | Good | |
| Lara, B et al., 2014 | YES | 1 | 1 | 1 | 1 | 1 | 0 | 1 | 1 | 1 | 1 | 10 | Excellent | |
| Carr A et al., 2008 | NO | 1 | 1 | 1 | 1 | 1 | 0 | 1 | 1 | 1 | 1 | 9 | Good | |

Note: “excellent” (10–11 points); “good” 179 (7–9 points); “fair” (5–6 points); and “poor” (0–4 points)

1. eligibility criteria were specified

2. subjects were randomly allocated to groups (in a crossover study, subjects were randomly allocated an order in which treatments were received)

3. allocation was concealed

4. the groups were similar at baseline regarding the most important prognostic indicators

5. there was blinding of all subjects

6. there was blinding of all therapists who administered the therapy

7. there was blinding of all assessors who measured at least one key outcome

8. measures of at least one key outcome were obtained from more than 85% of the subjects initially allocated to groups

9. all subjects for whom outcome measures were available received the treatment or control condition as allocated or, where this was not the case, data for at least one key outcome was analyzed by “intention to treat”

10. the results of between-group statistical comparisons are reported for at least one key outcome

11. the study provides both point measures and measures of variability for at least one key outcome

**Supplementary Material Appendix S3 (GRADE assessment)**

| **Outcome** | **K** | **Certainty of Evidence Assessment** | | | | | **Hedge’s g [95% CI] *** | **GRADE†** |
| --- | --- | --- | --- | --- | --- | --- | --- | --- |
|  |  | **Risk of Bias** | **Inconsistency** | **Indirectness** | **Imprecision** | **Others** |  |  |

| Primary Outcome | | | | | | | | | | | | | | | | | | | | | | | | |
| --- | --- | --- | --- | --- | --- | --- | --- | --- | --- | --- | --- | --- | --- | --- | --- | --- | --- | --- | --- | --- | --- | --- | --- | --- |
| Exercise performance | | | | K = 43 | | | Serious | | | Serious | | | Not serious | | | Not serious | | Publication bias | | | 0.28 [0.14, 0.43] ***** | | | ⨁⨁◯◯ Low |
| HR | | | | K = 6 | | | Serious | | | Serious | | | Not serious | | | Serious | | None | | 0.23 [-0.07, 0.54] | | | ⨁◯◯◯ Very low | |
| BLC | | | | K = 13 | | | Not serious | | | Not serious | | | Serious | | | Not serious | | Publication bias | | 0.50 [0.22, 0.79] ***** | | | ⨁⨁◯◯ Low | |
| RPE | | | | K = 12 | | | Not serious | | | Serious | | | Serious | | | Serious | | None | | -0.11 [-0.37, 0.15] | | | ⨁◯◯◯ Very low | |
| Glucose | | | | K = 4 | | | Not serious | | | Serious | | | Not serious | | | Not serious | | None | | | 0.56 [0.17, 0.96] ***** | | | ⨁⨁◯◯ Low |
| Sex | | | | | | | | | | | | | | | | | | | | | | | | |
| Male | | | | K = 35 | | | Not serious | | | Serious | | | Not serious | | | Not serious | | Publication bias | | 0.30 [0.13, 0.48] ***** | | | ⨁⨁◯◯ Low | |
| Female | | | | K = 6 | | | Not serious | | | Serious | | | Serious | | | Not serious | | None | | 0.34 [0.01, 0.66] ***** | | | ⨁⨁◯◯ Low | |
| mix | | | | K = 2 | | | Not serious | | | Serious | | | Serious | | | Serious | | None | | -0.06 [-0.30, 0.17] | | | ⨁◯◯◯ Very low | |
| Training status | | | | | | | | | | | | | | | | | | | | | | | |  |
| Recreationally active | | K = 23 | | | Serious | | | Not serious | | | Serious | | | Not serious | | Publication bias | | 0.21 [-0.02, 0.44] | | | ⨁⨁◯◯ Low | | |  |
| Trained | | K = 14 | | | Not serious | | | Not serious | | | Not serious | | | Not serious | | Publication bias | | 0.44 [0.27, 0.61] ***** | | | ⨁⨁⨁◯ Moderate | | |  |
| Well-trained | | K = 6 | | | Not serious | | | Serious | | | Serious | | | Serious | | None | | 0.06 [-0.11, 0.23] | | | ⨁◯◯◯ Very low | | |  |
| Caffeine dose | | | | | | | | | | | | | | | | | | | | | | | | |
| 0–3 | | K = 11 | | | Not serious | | | Serious | | | Serious | | | Not serious | | | None | | 0.23 [0.15, 0.51] ***** | | | ⨁⨁◯◯ Low | | |
| 3–6 | | K = 32 | | | Serious | | | Not serious | | | Not serious | | | Not serious | | | Publication bias | | 0.38 [0.07, 0.49] ***** | | | ⨁⨁◯◯ Low | | |
| Caffeine form | | | | | | | | | | | | | | | | | | | | | | | |  |
| Capsules | | K = 29 | | | Not serious | | | Serious | | | Not serious | | | Not serious | | Publication bias | | 0.33 [0.08, 0.57] ***** | | | ⨁⨁◯◯ Low | | |  |
| Beverage | | K = 11 | | | Serious | | | Serious | | | Not serious | | | Not serious | | None | | 0.32 [0.13, 0.51] ***** | | | ⨁⨁◯◯ Low | | |  |
| Gum | | K = 3 | | | Not serious | | | Serious | | | Not serious | | | Serious | | None | | 0.01 [-0.20, 0.22] | | | ⨁⨁◯◯ Low | | |  |
| Interval type | | | | | | | | | | | | | | | | | | | | | | | |  |
| RSE | | K = 27 | | | Not serious | | | Not serious | | | Serious | | | Not serious | | Publication bias | | 0.25 [0.08, 0.42] ***** | | | ⨁⨁◯◯ Low | | |  |
| ISE | | K = 10 | | | Serious | | | Serious | | | Not serious | | | Serious | | Publication bias | | 0.33 [0.14, 0.52] ***** | | | ⨁◯◯◯ Very low | | |  |
| Short intervals | | K = 6 | | | Not serious | | | Serious | | | Not serious | | | Serious | | None | | 0.60 [-0.51, 1.71] ***** | | | ⨁⨁◯◯ Low | | |  |

**S****upplementary Material Appendix** **S4 (A sensitivity analysis)**


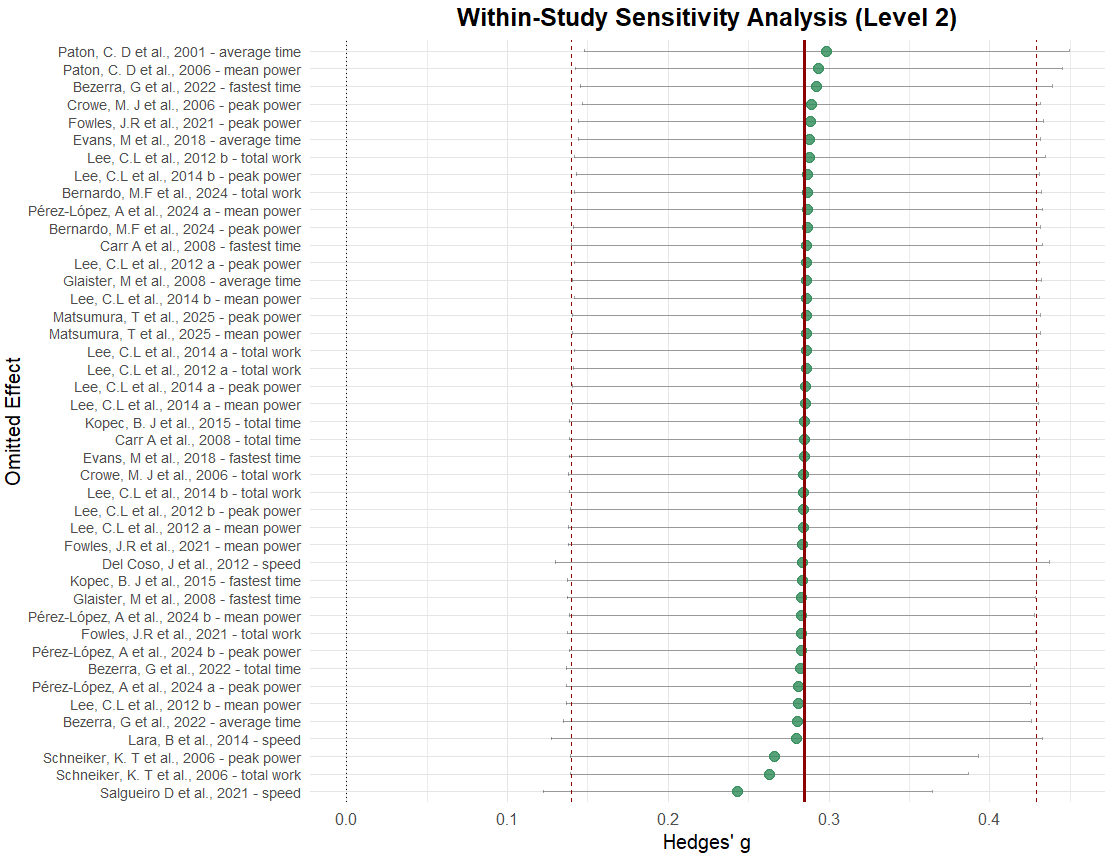

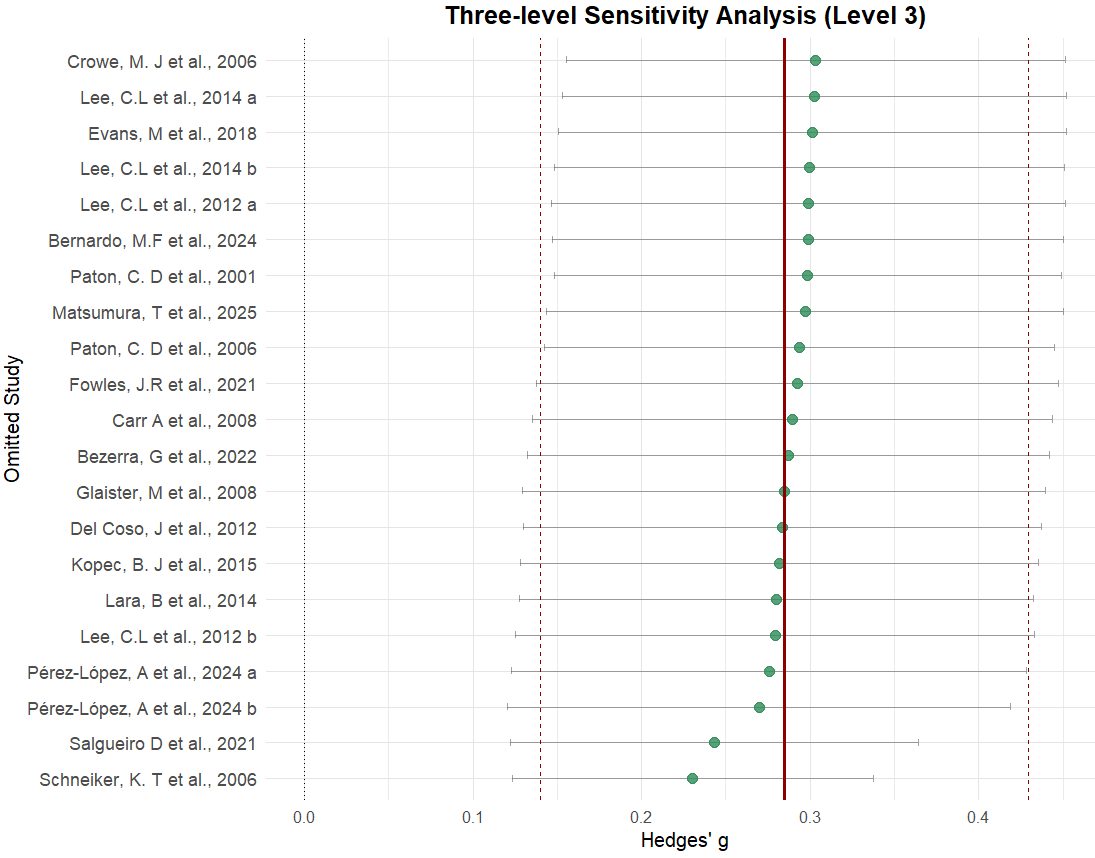


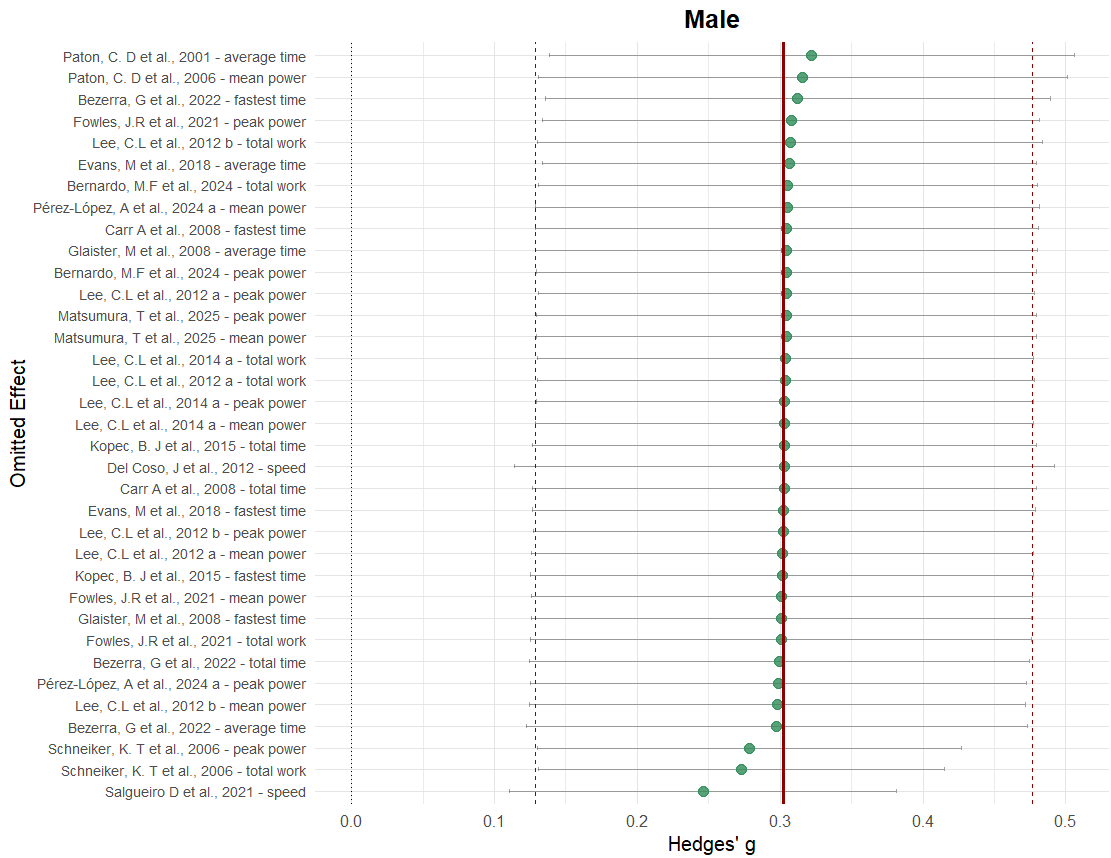

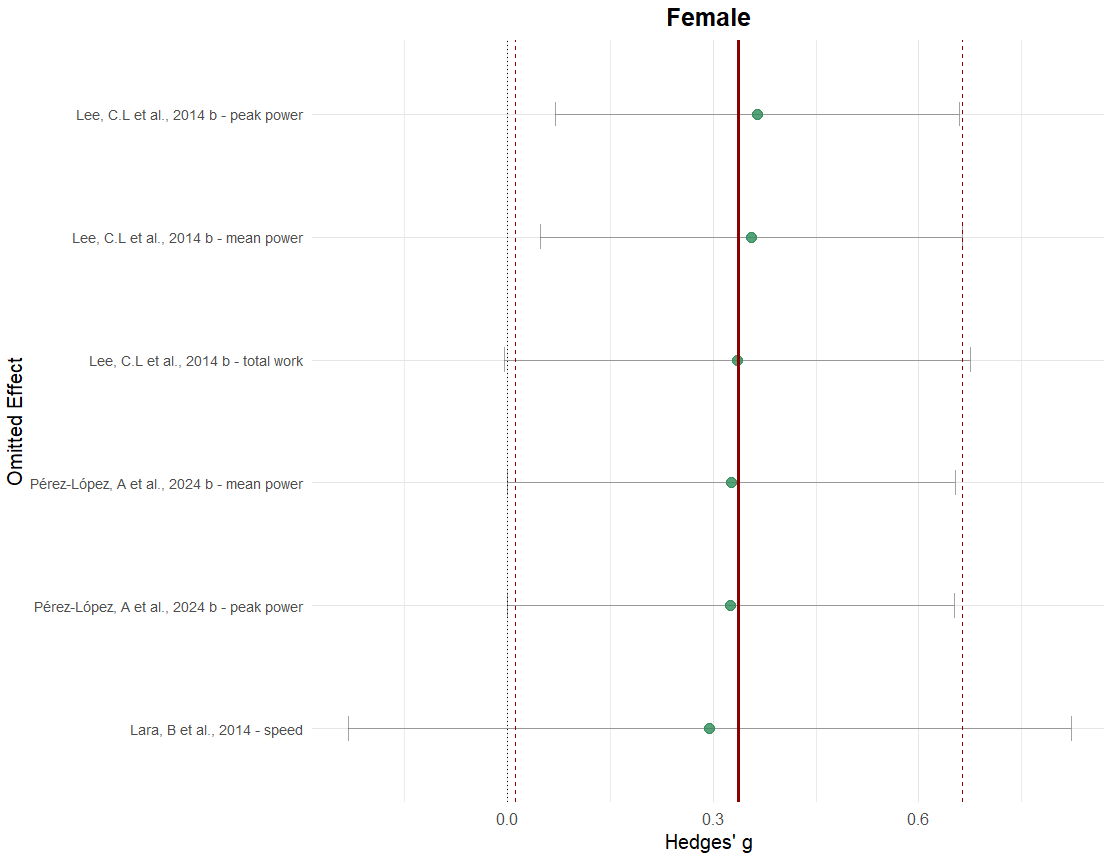

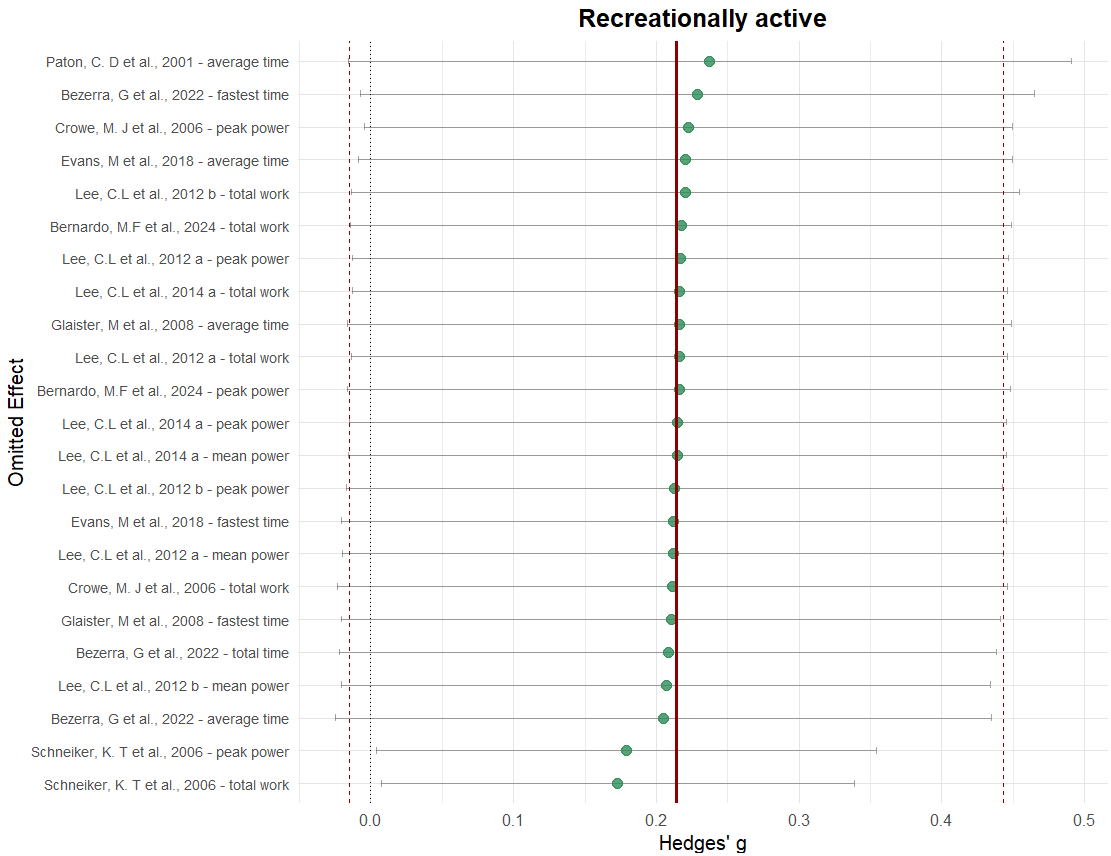

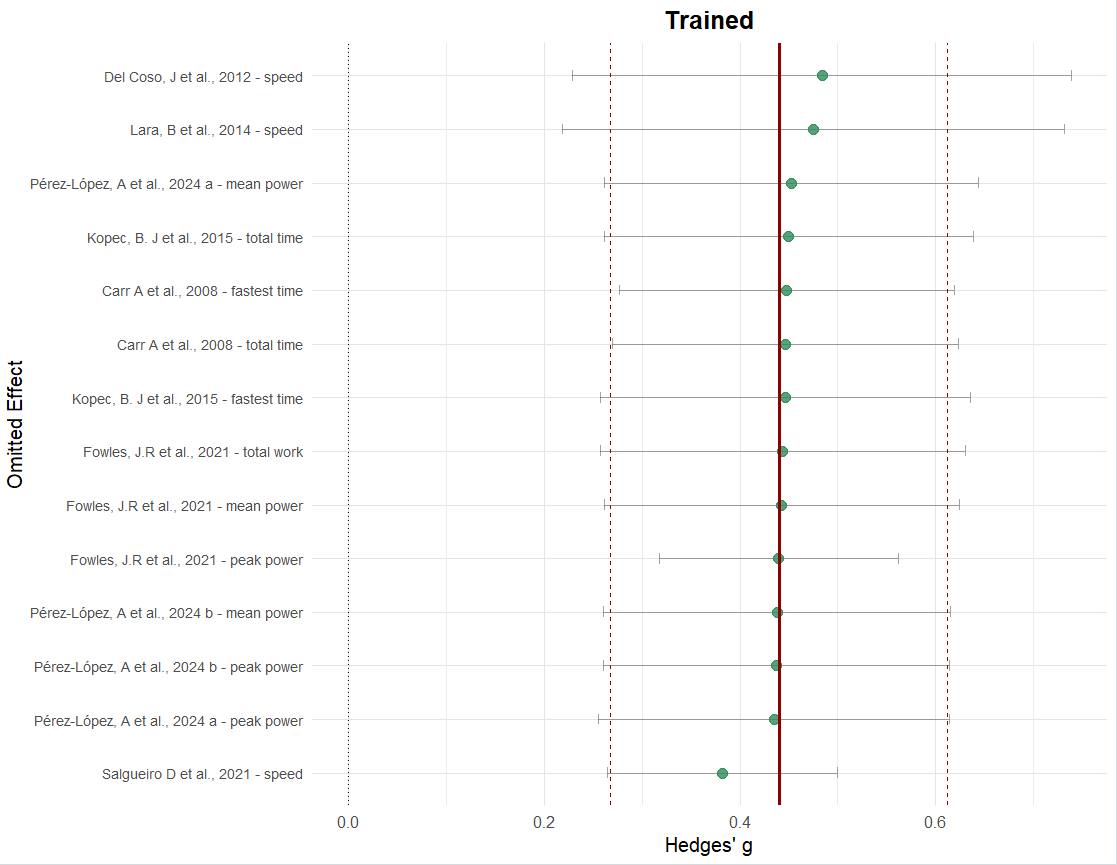

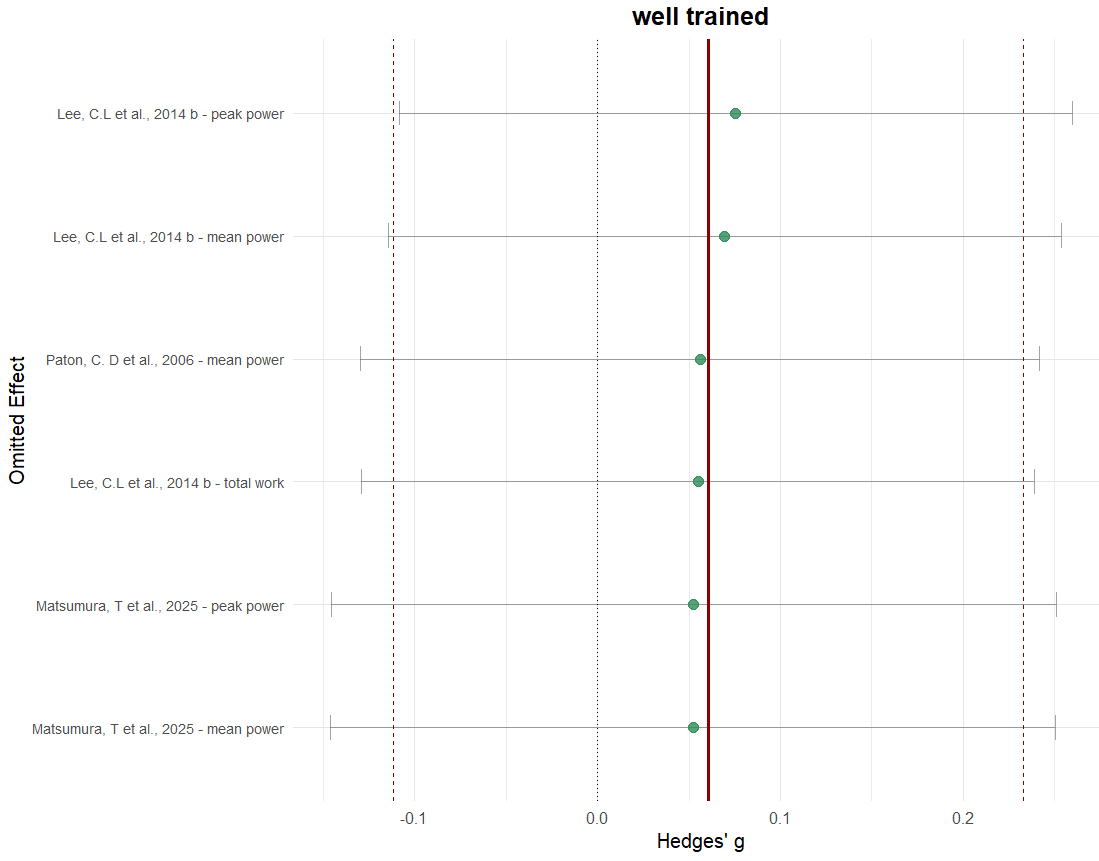


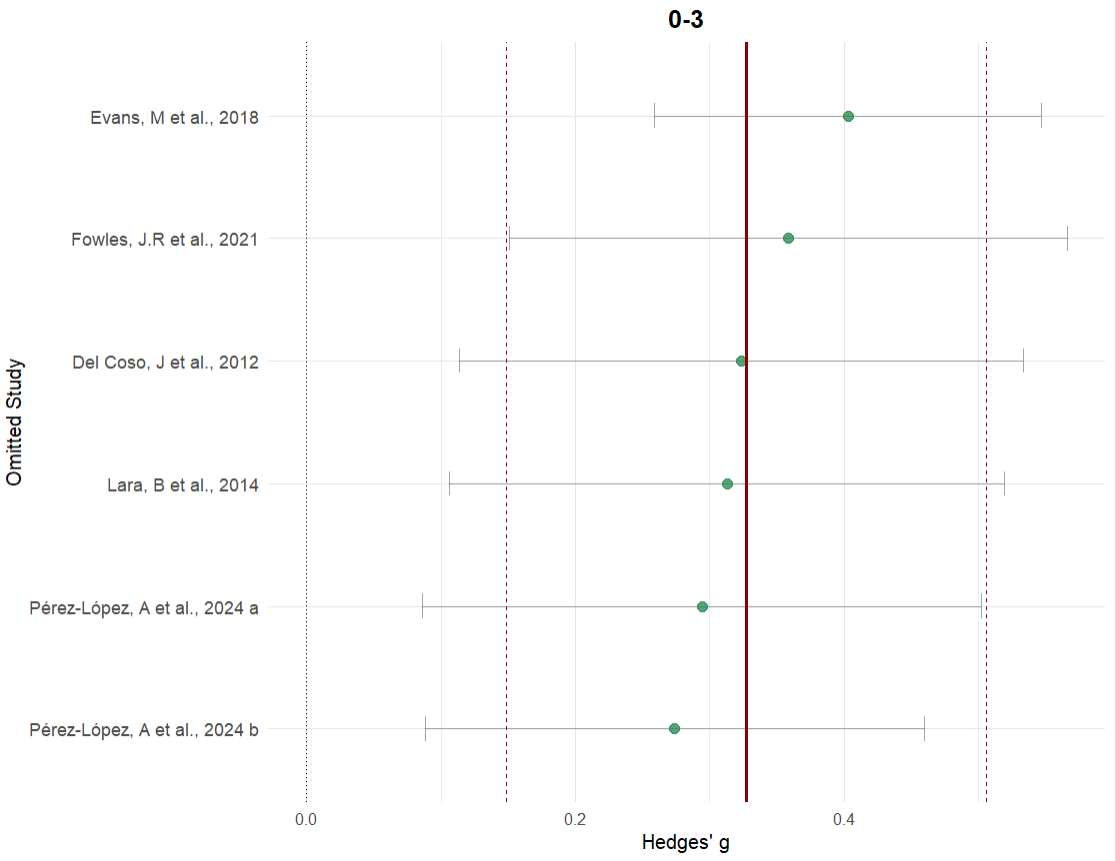

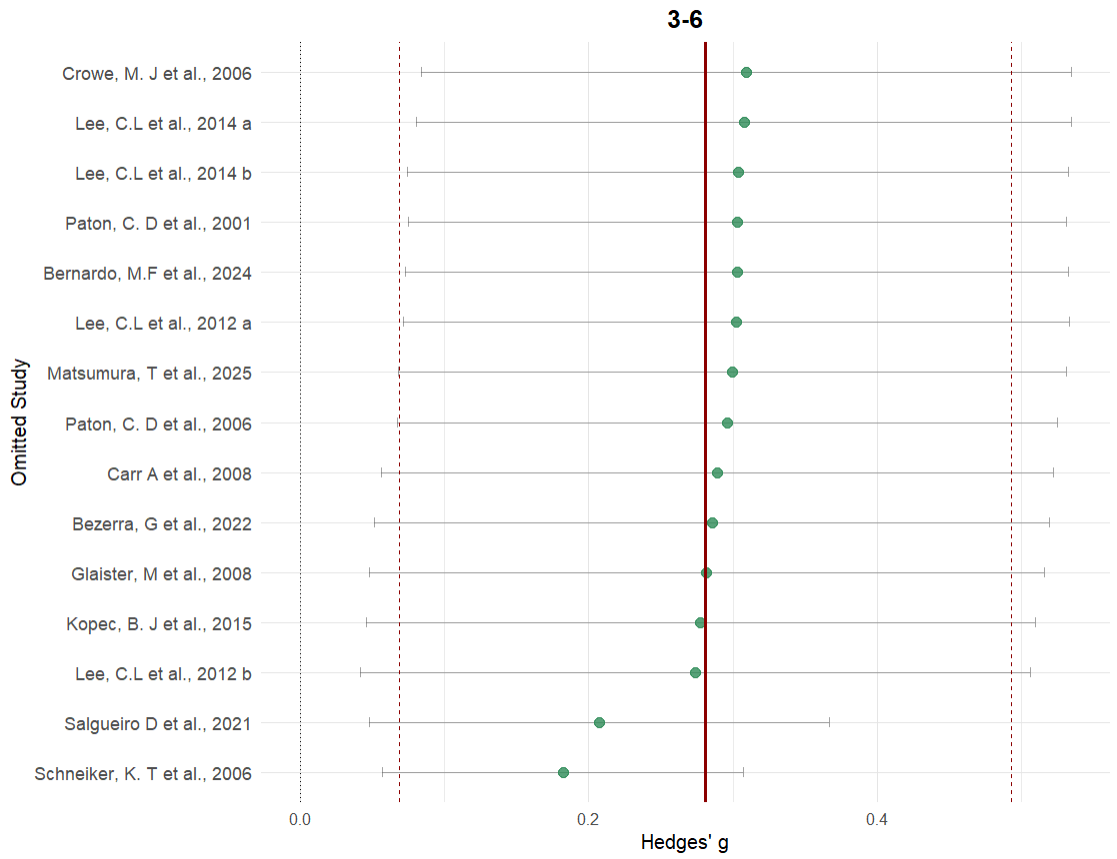

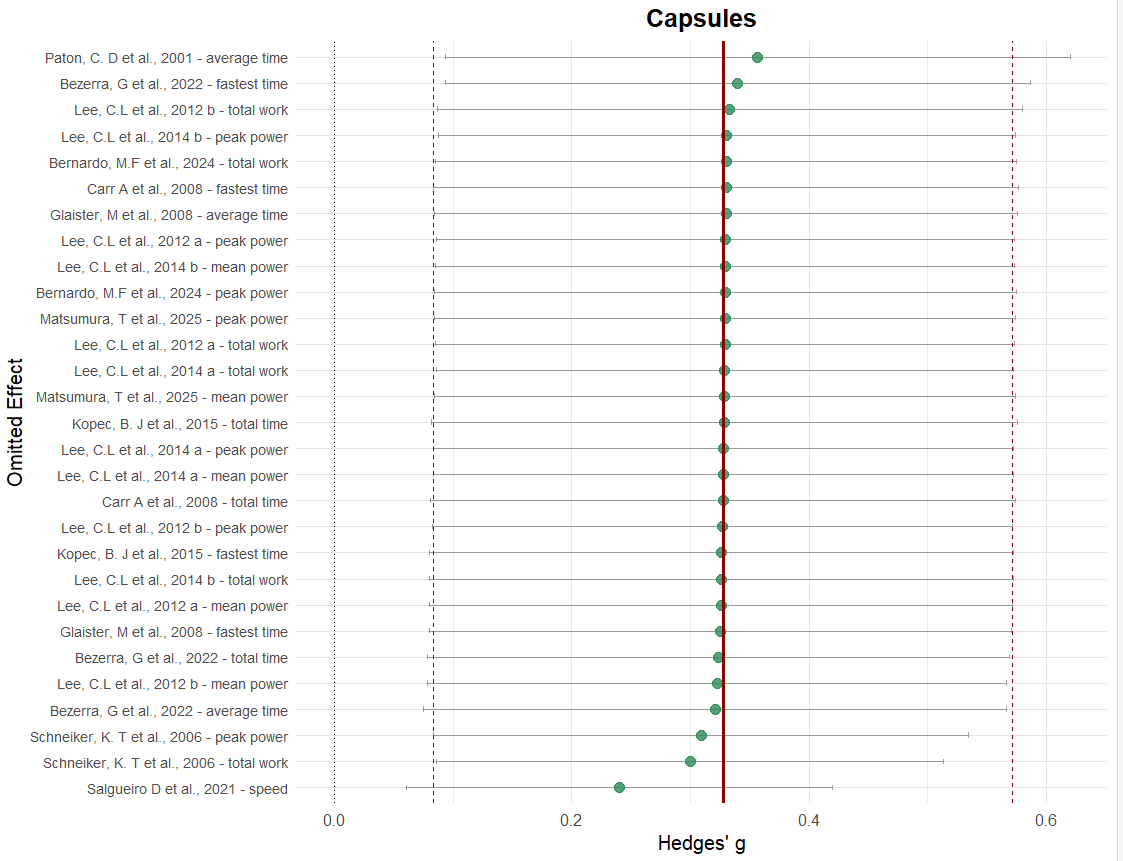

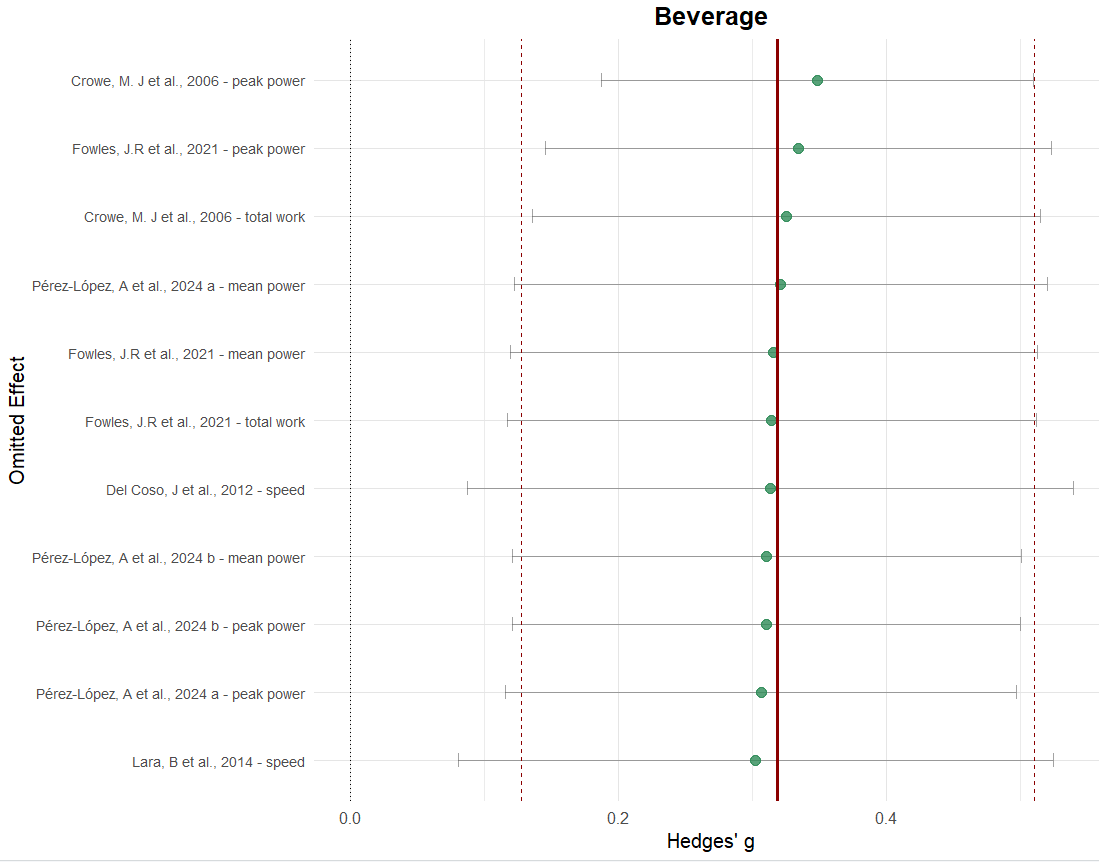

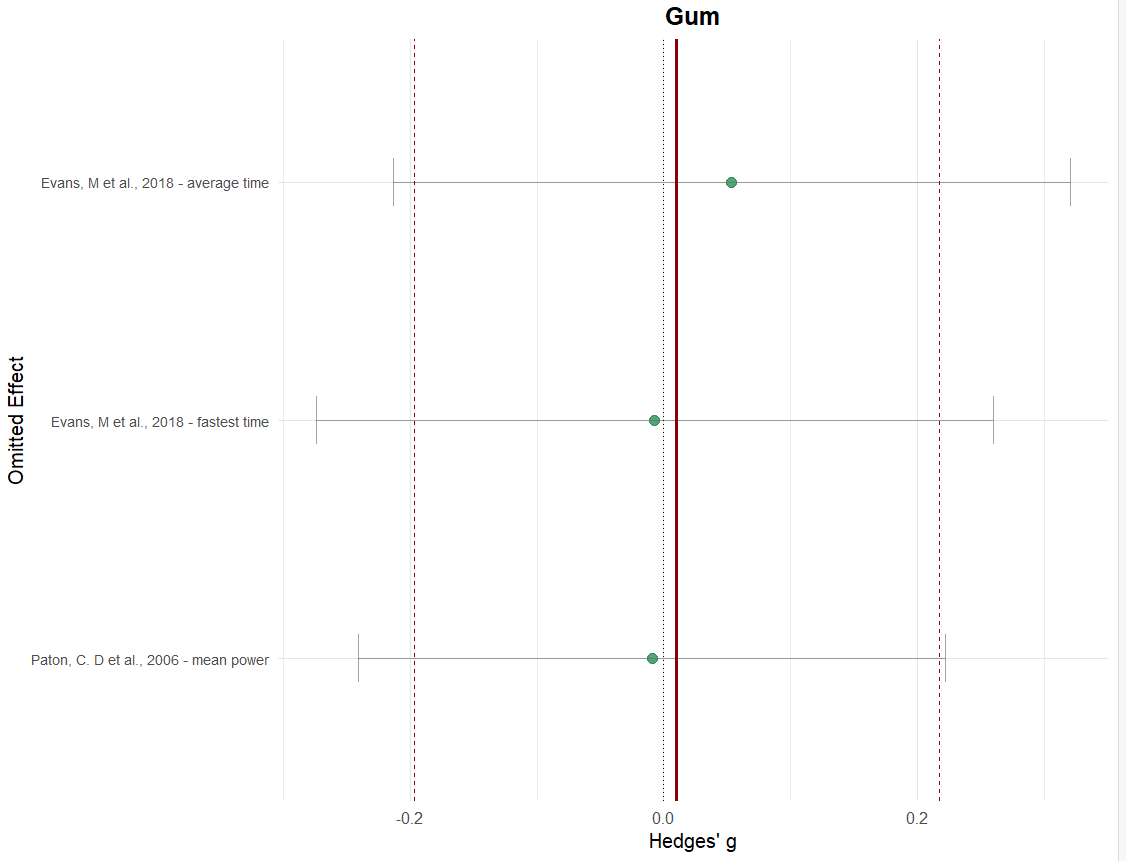


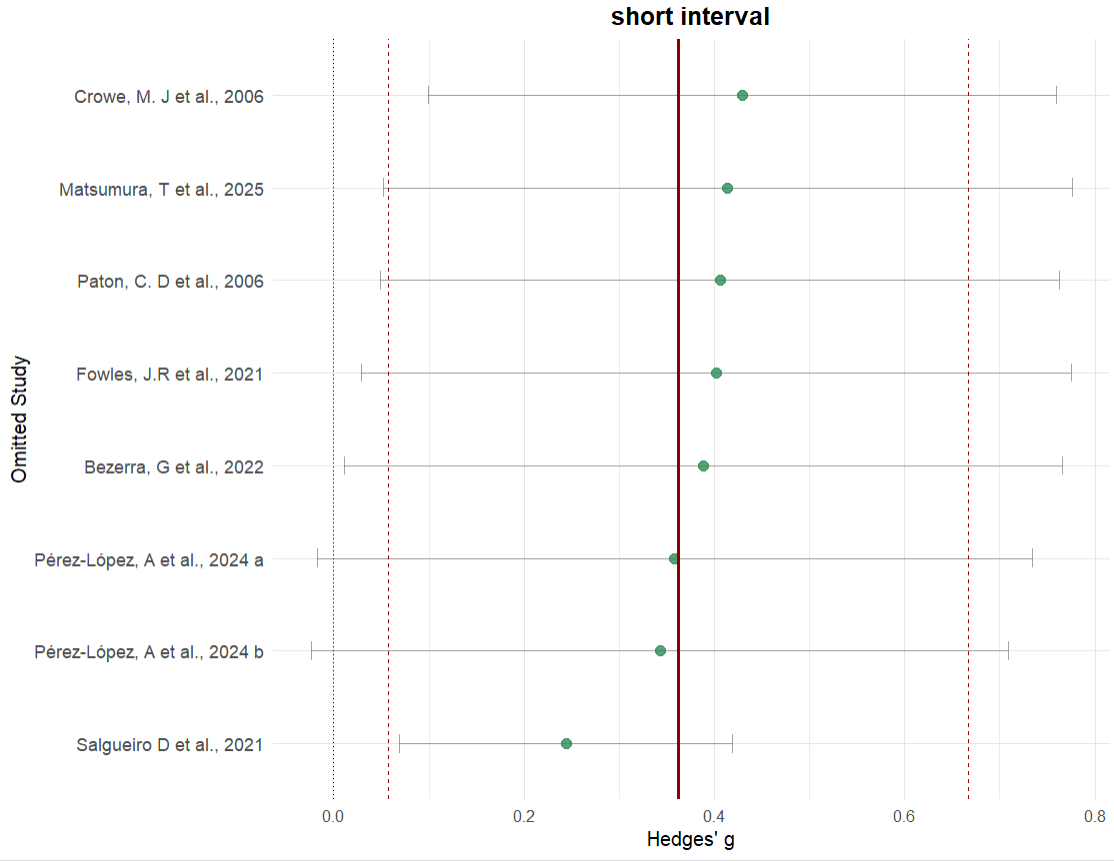

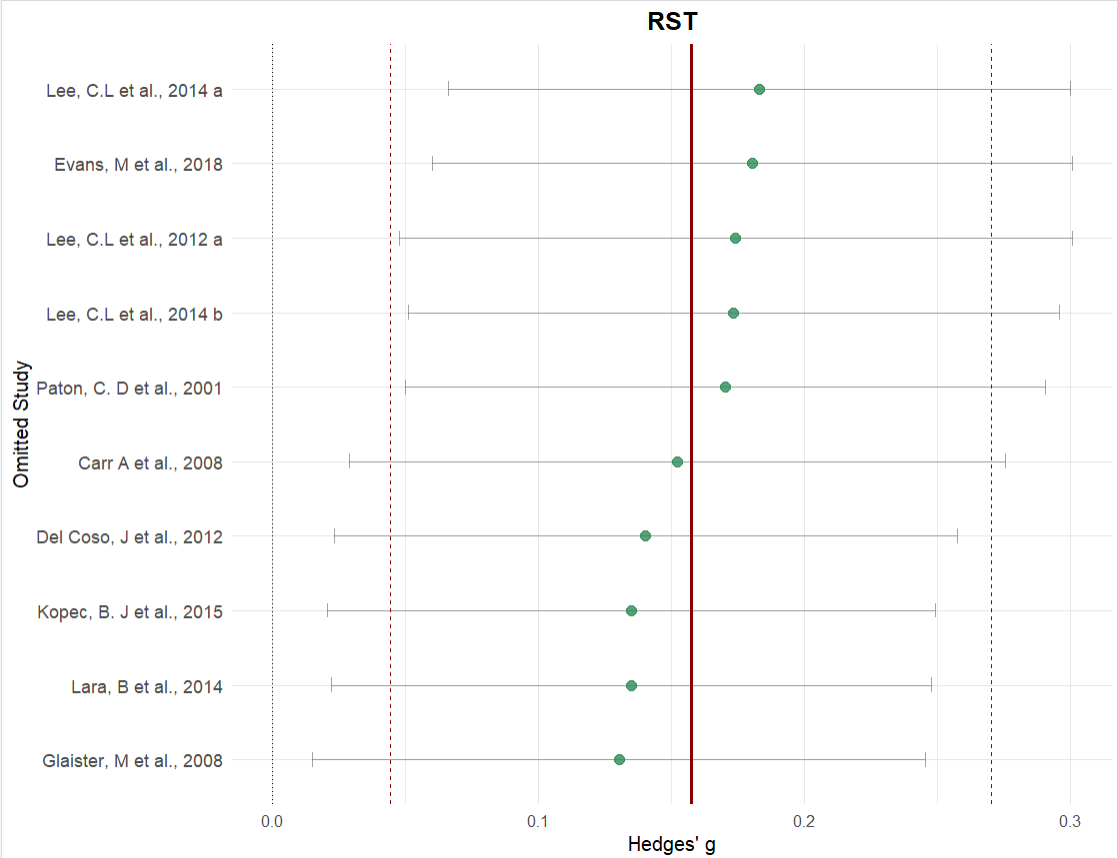

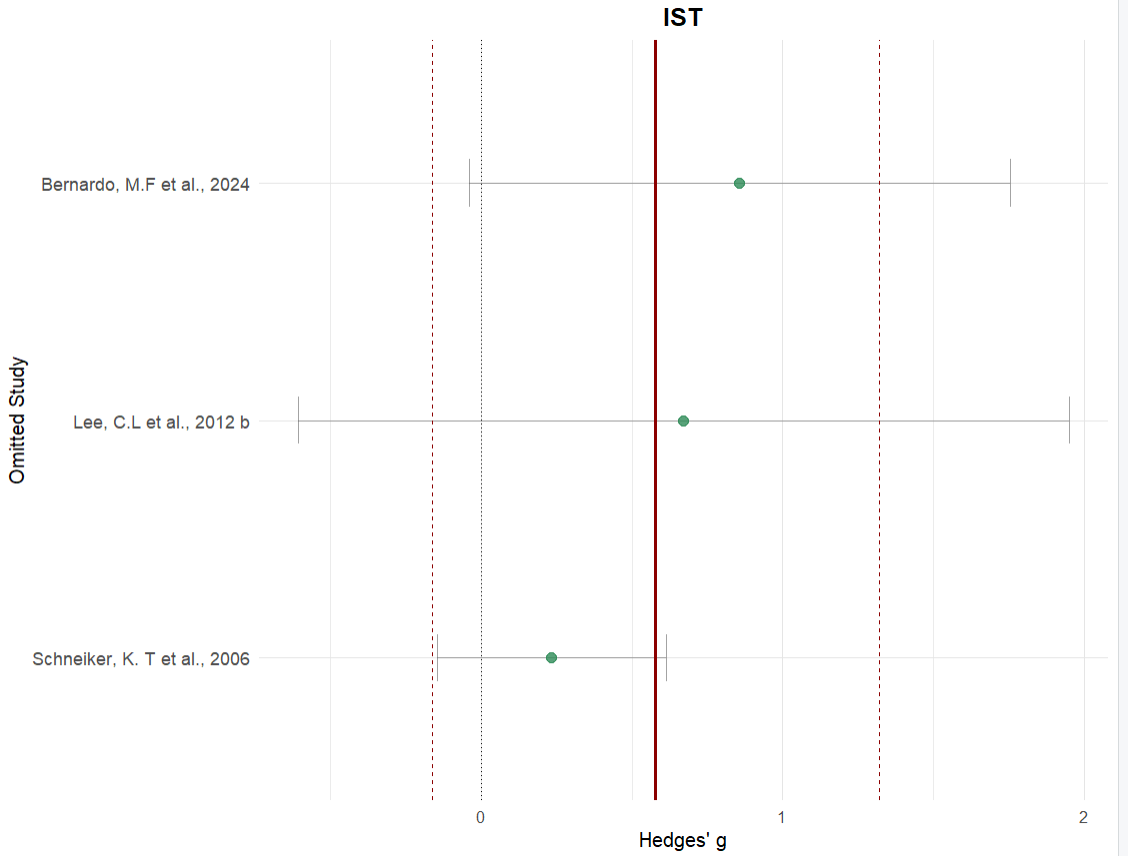


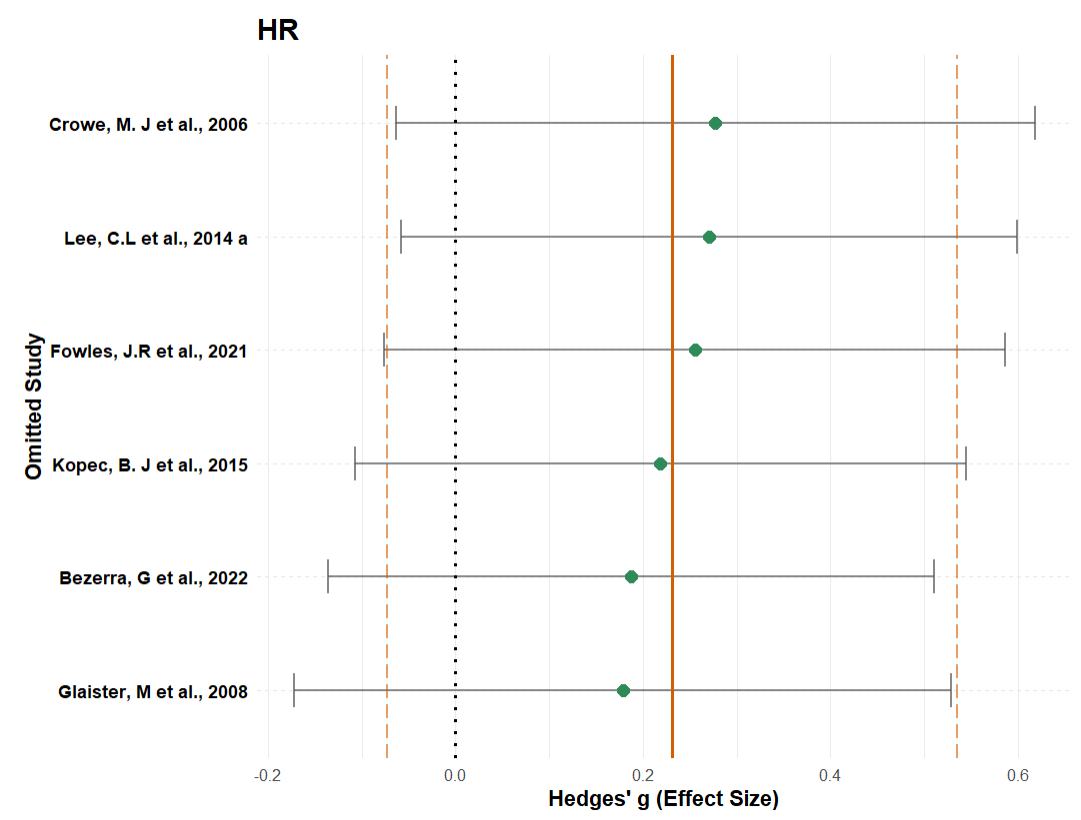

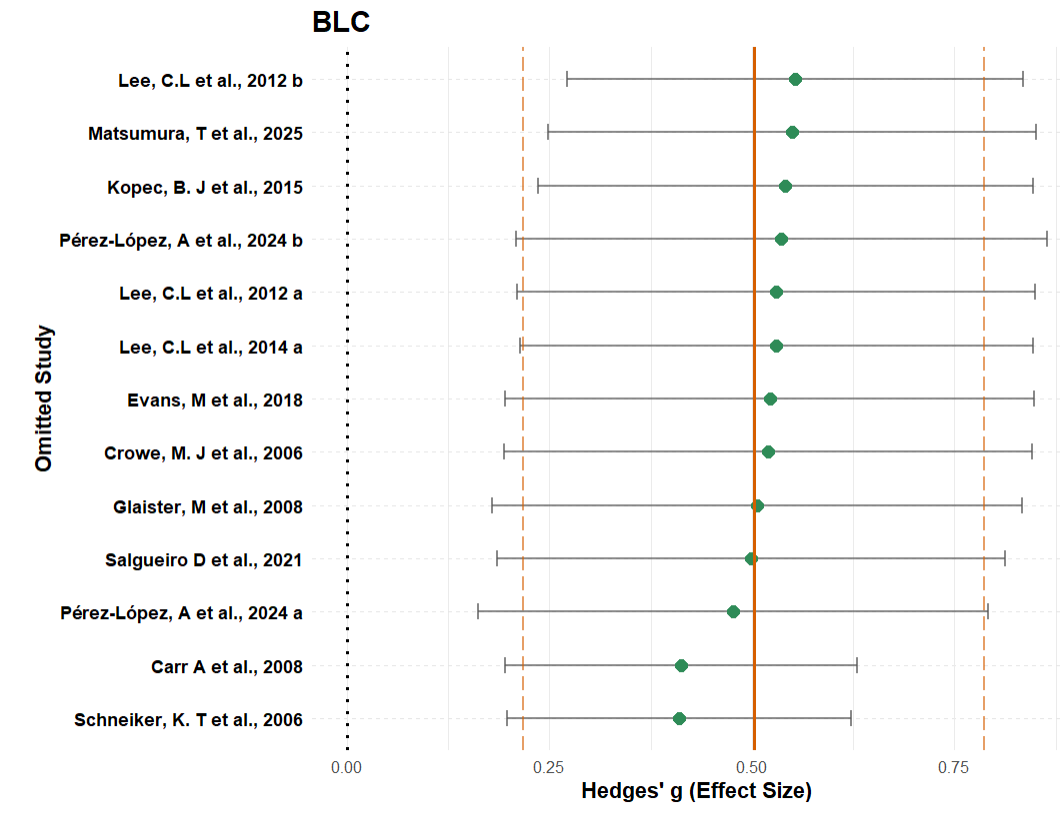

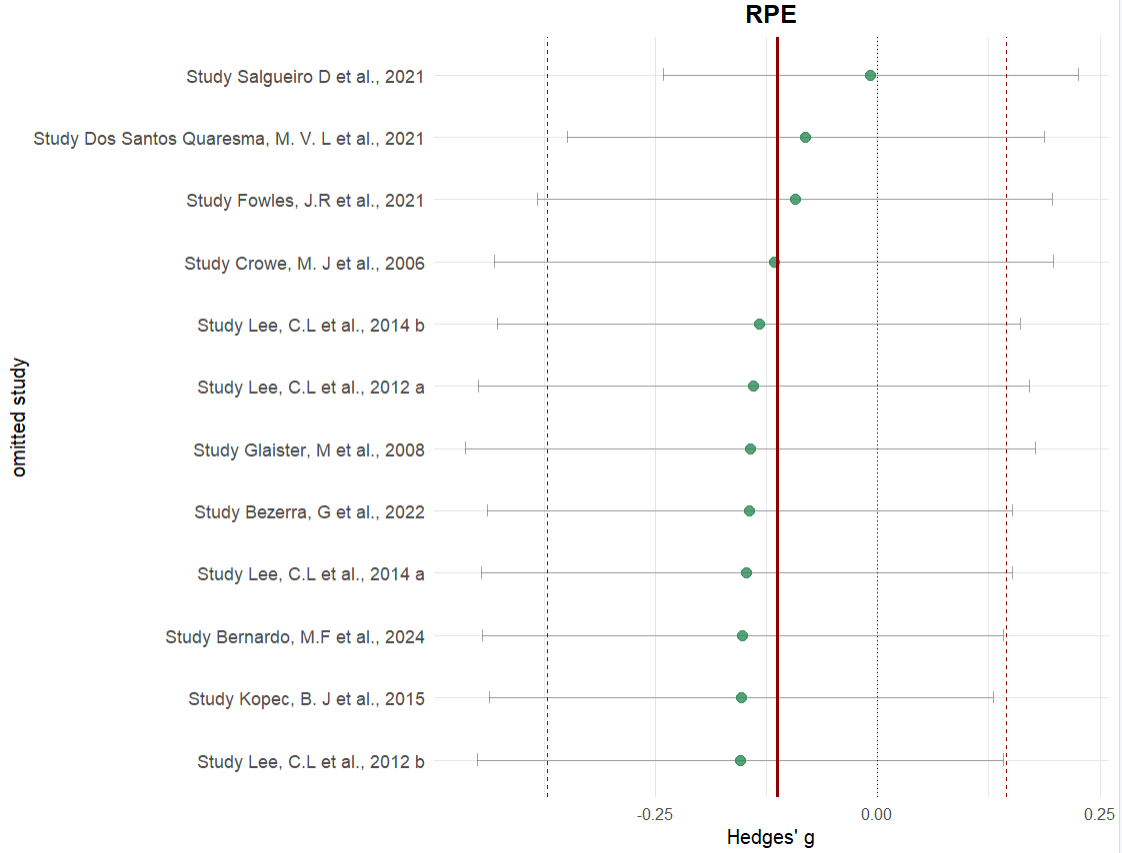

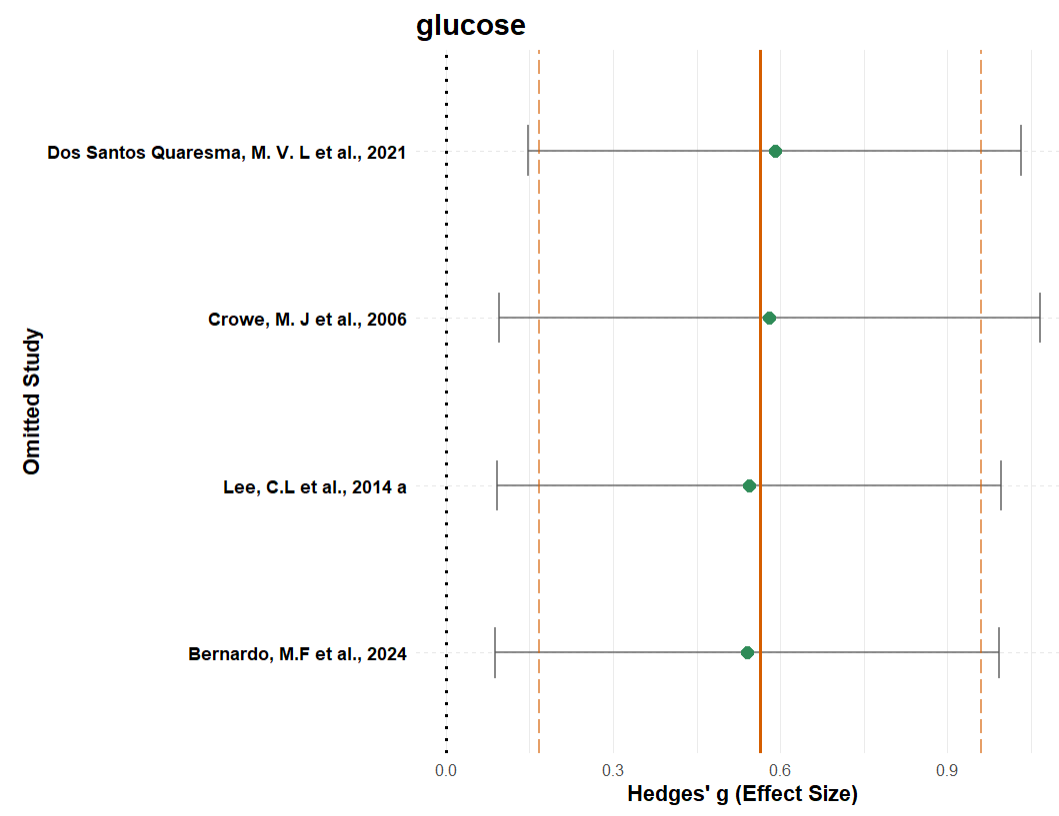


**Supplementary Material Appendix S5 (Funnel plot)**


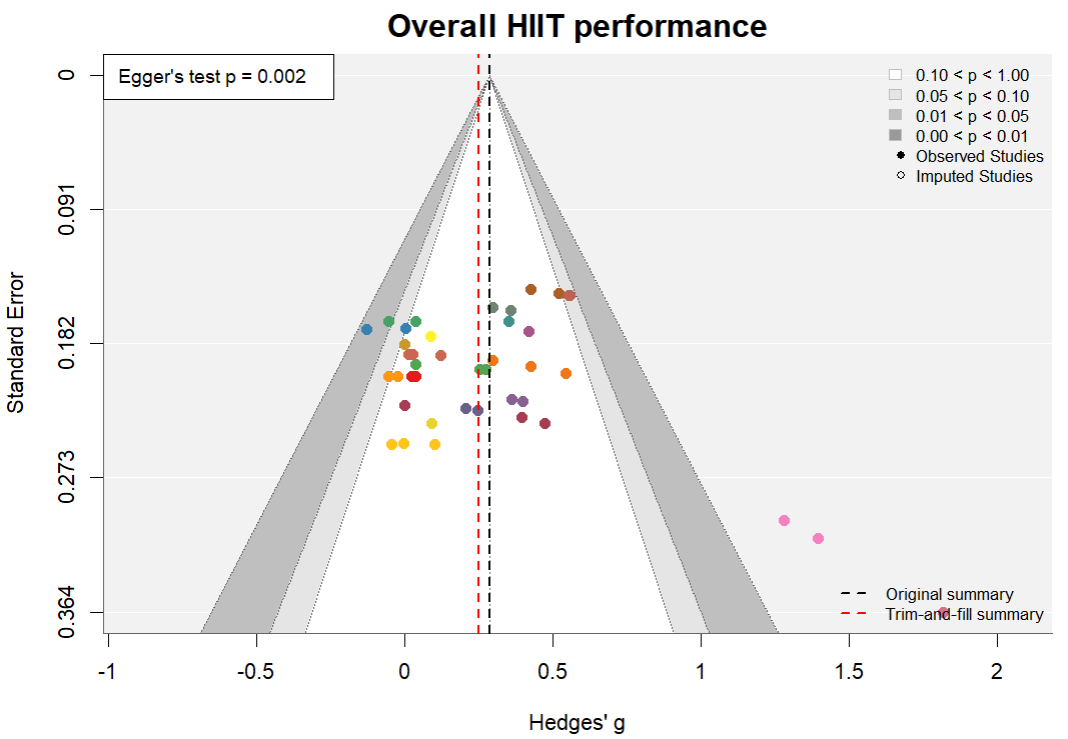


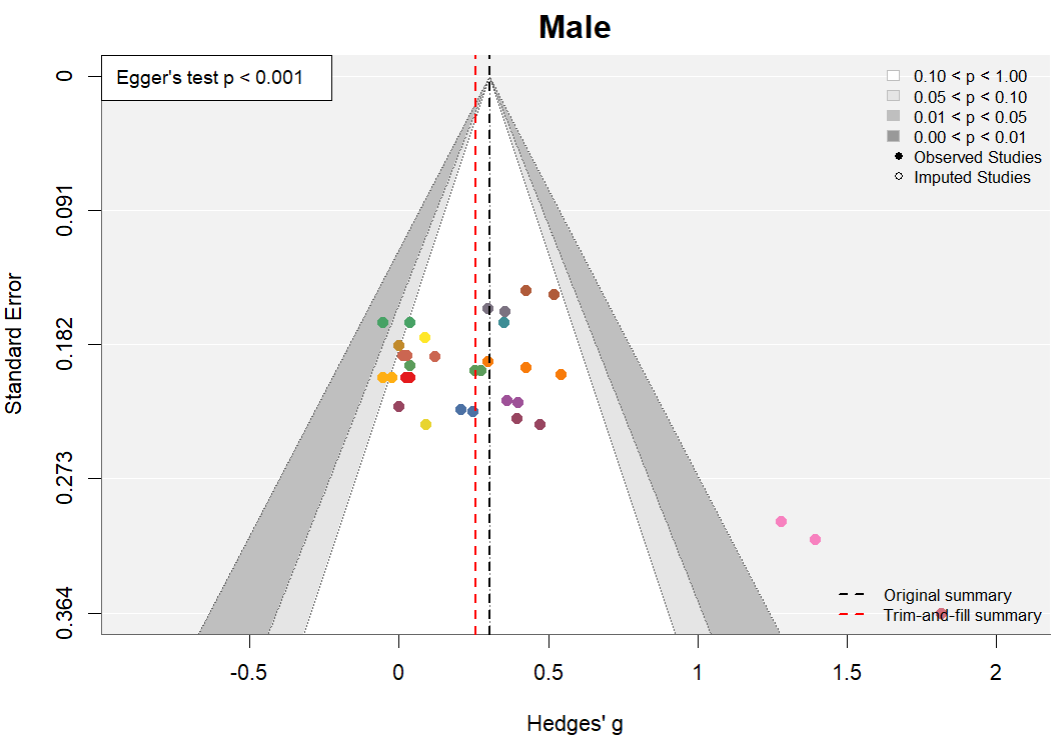

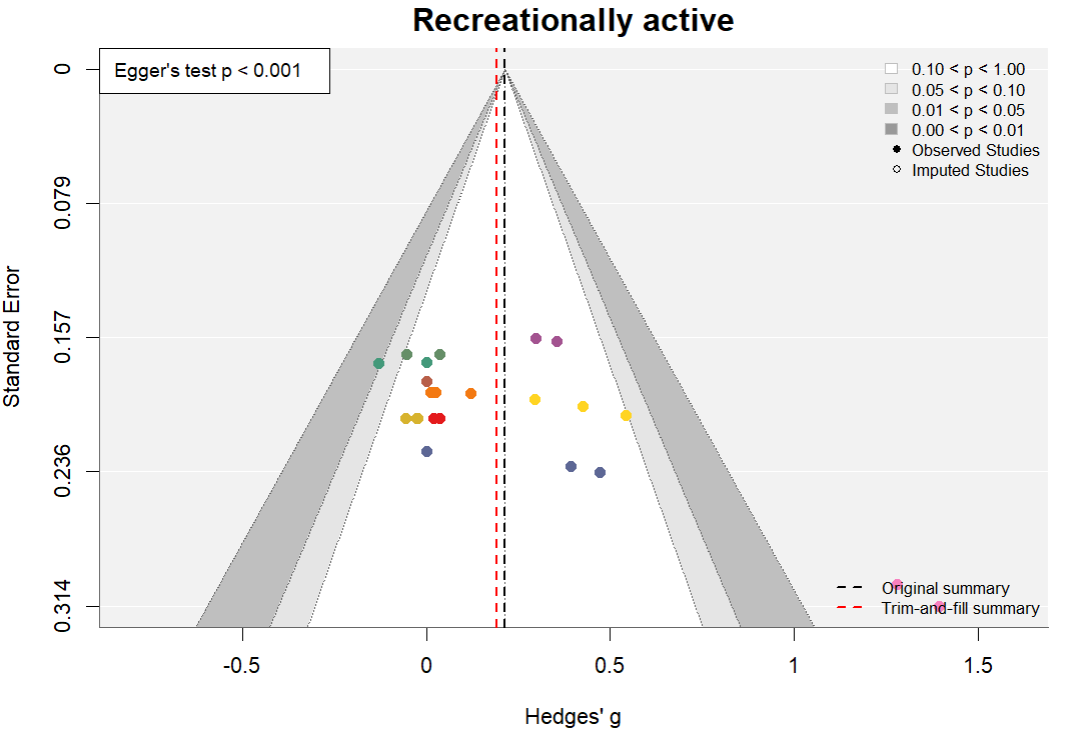

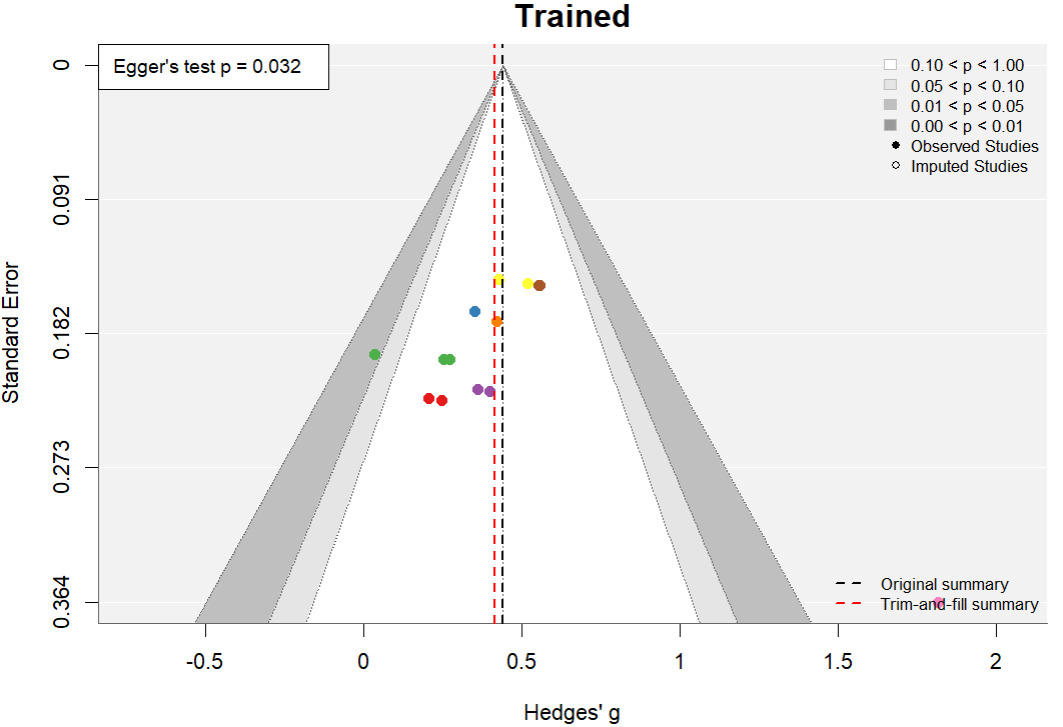


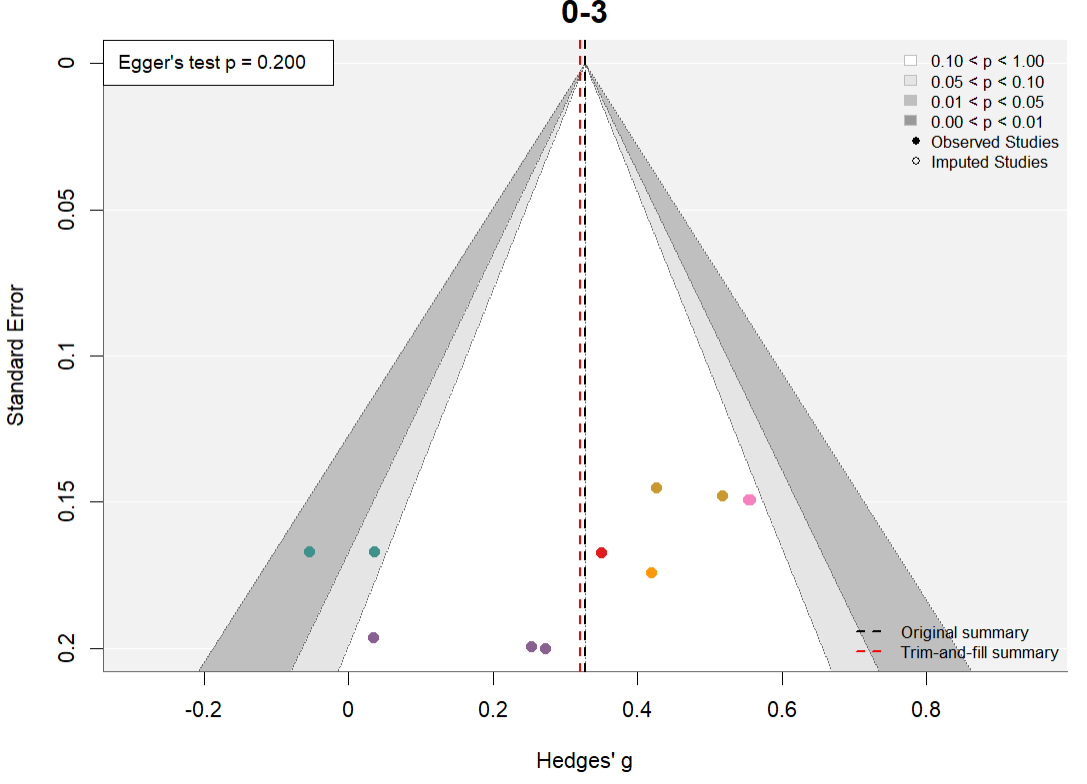

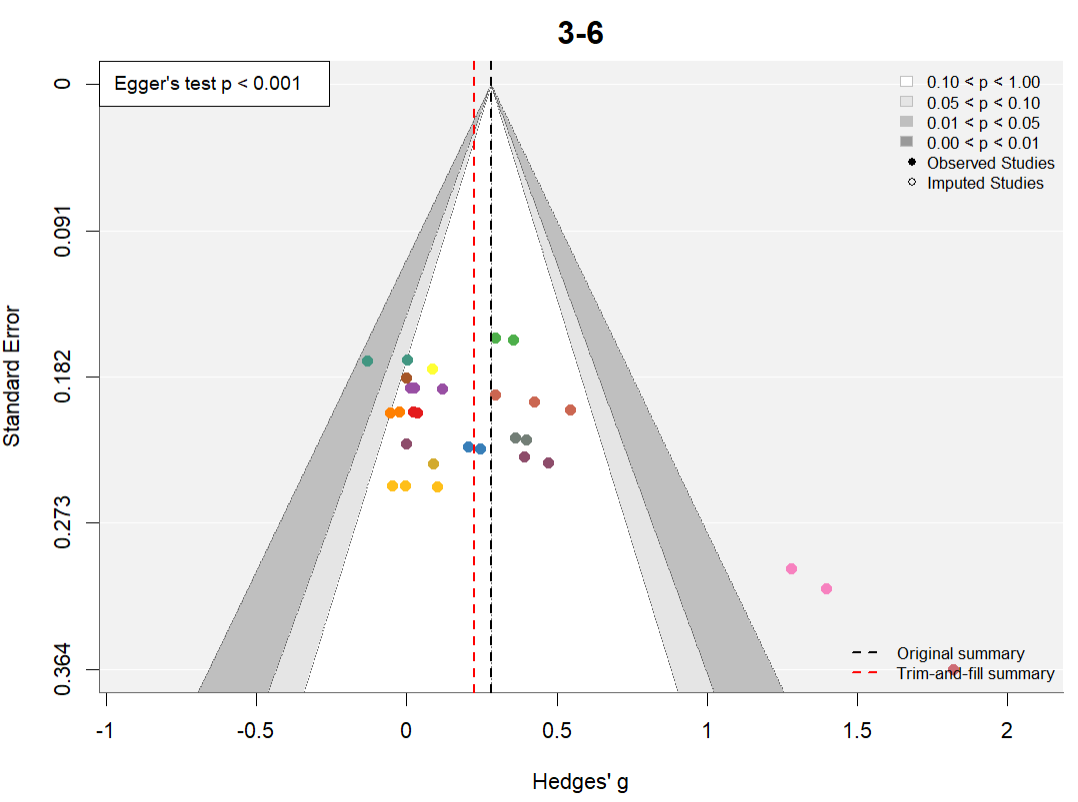


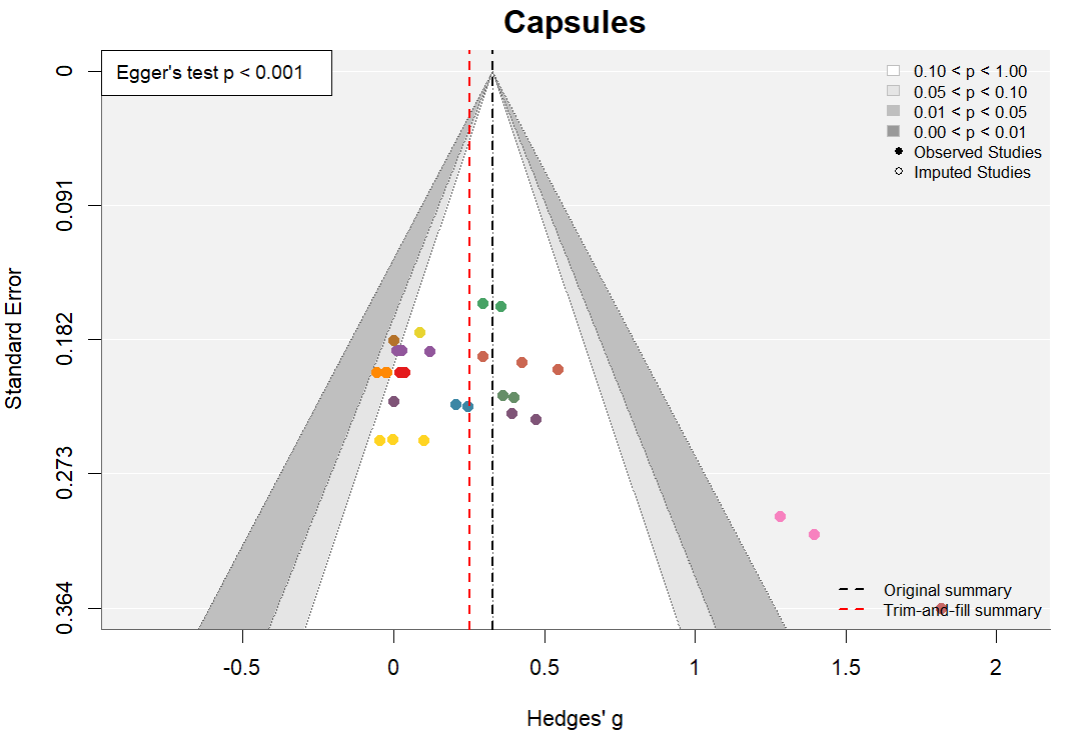

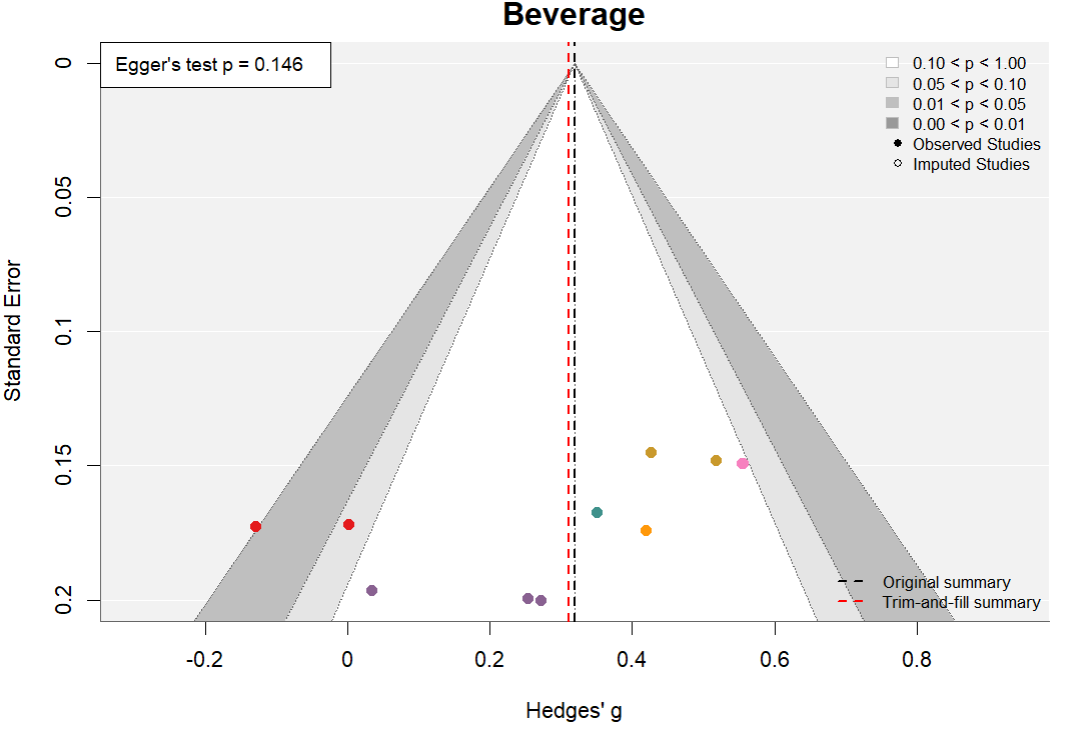


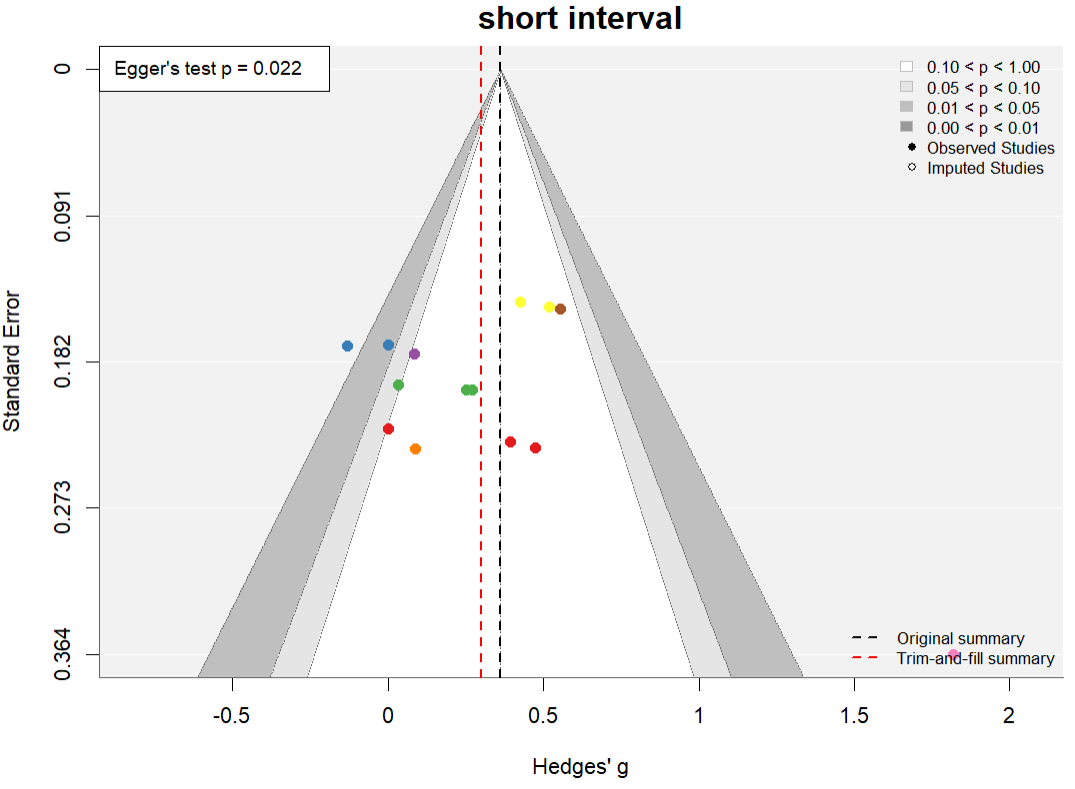

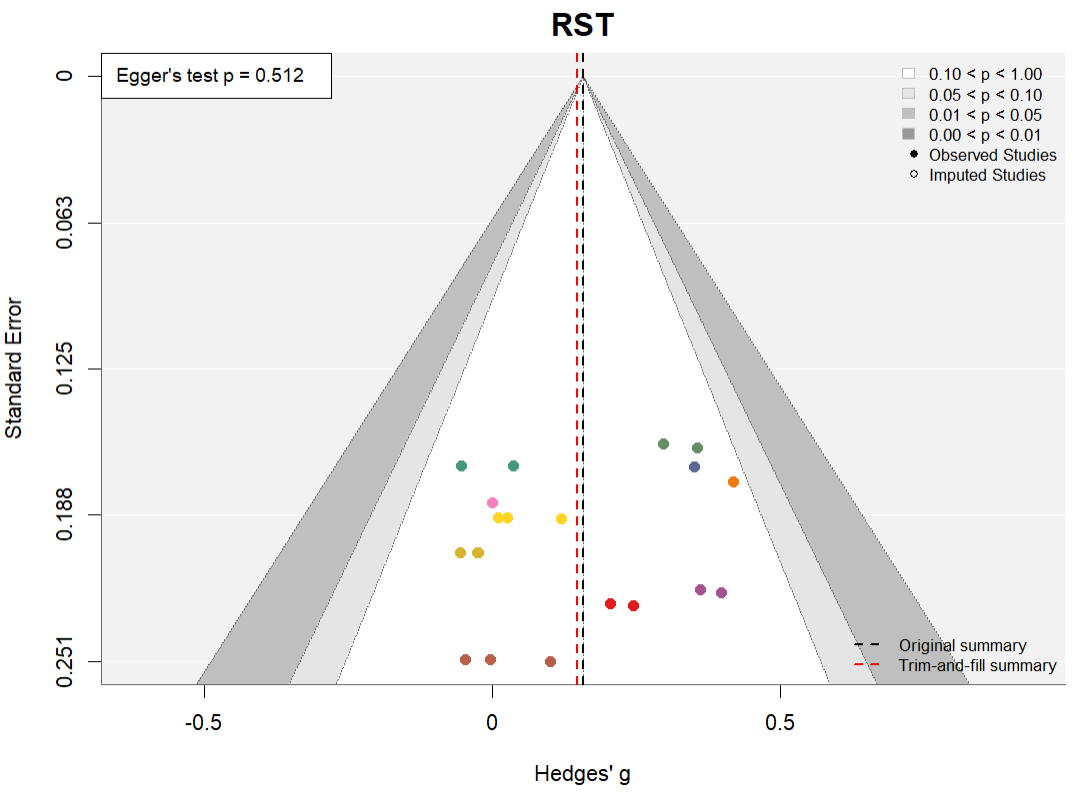


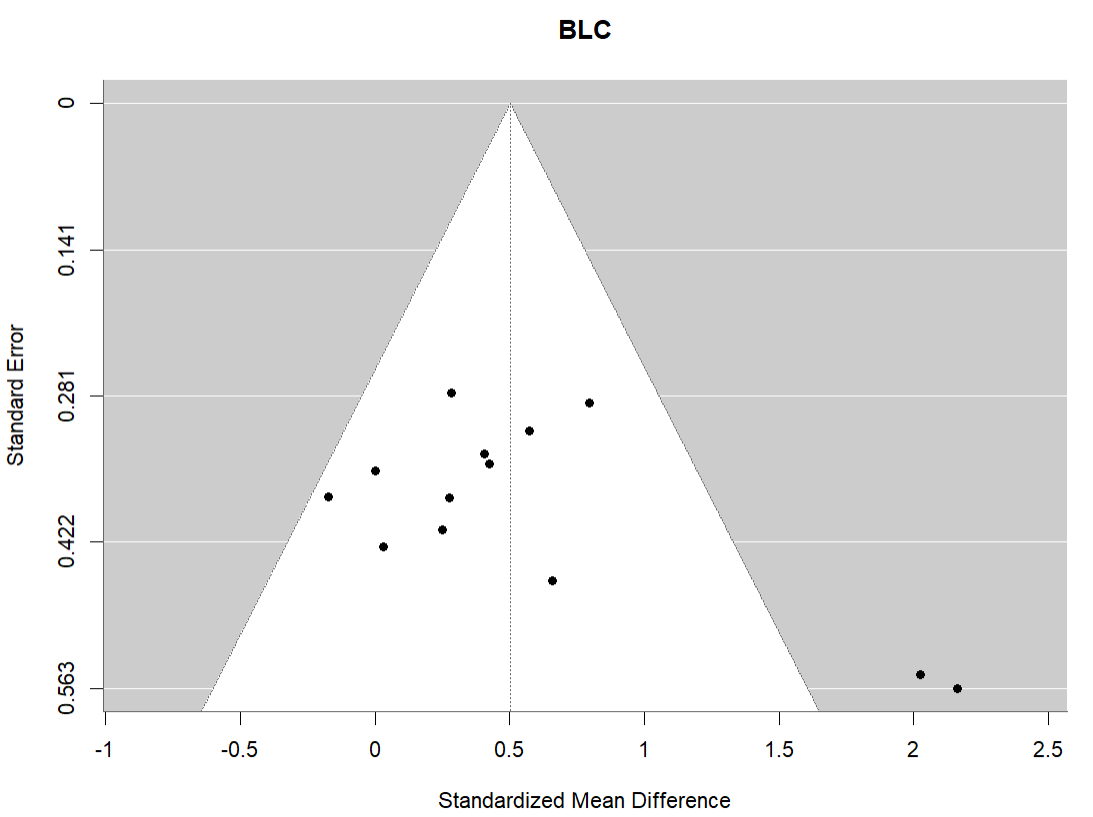

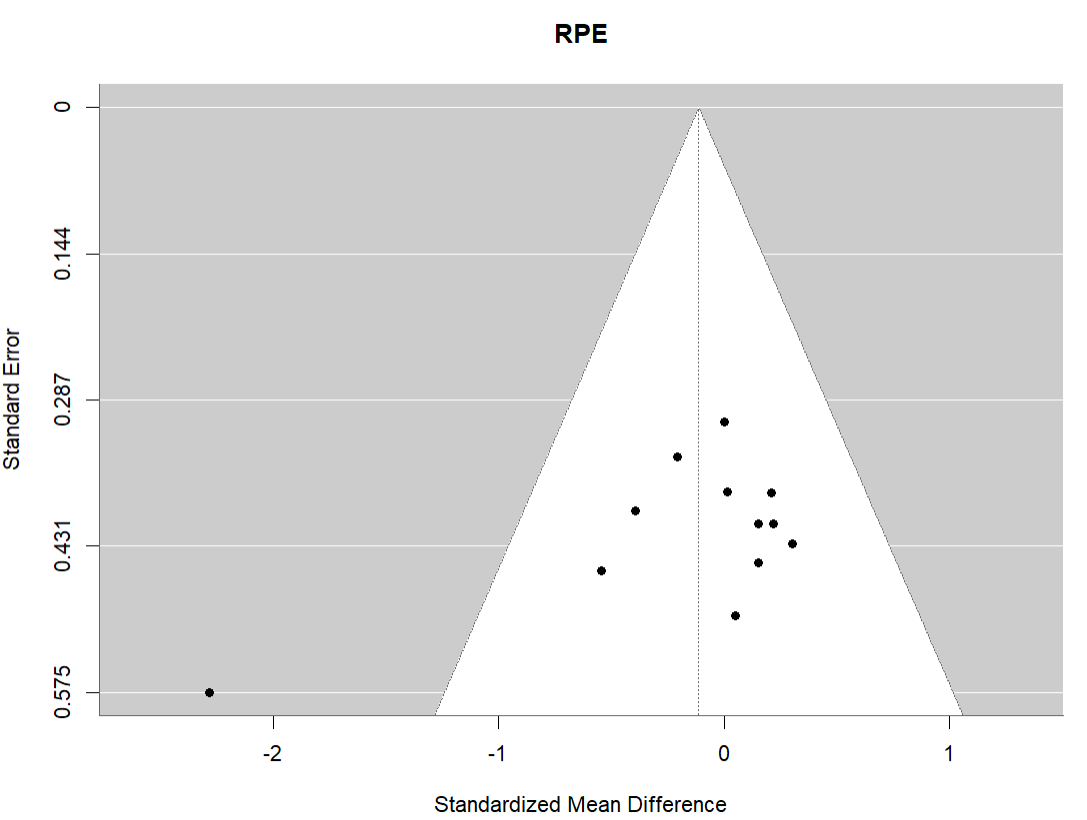


**Fig 8** Funnel plot of standard mean difference against standard error. HR, heart rate; BLC, blood lactate concentration; RPE, ratings of perceived exertion. RST, repeated sprint training SIT, sprints interval training.

**Supplementary Material Appendix S6 (Risk of bias summary)**
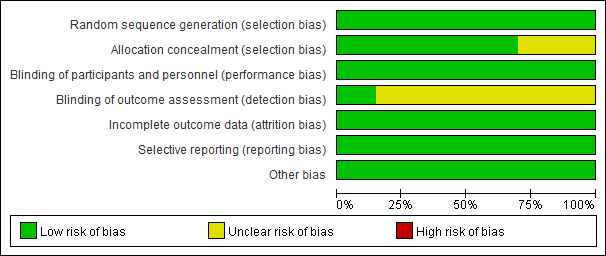

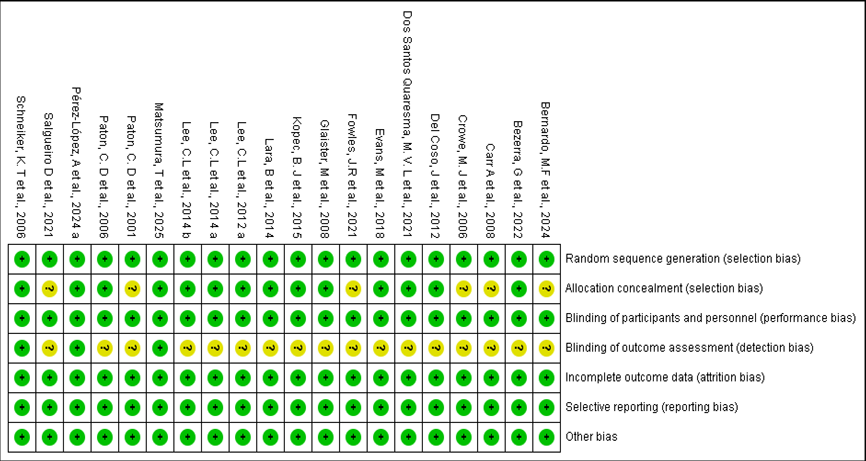


Risk of bias summary of included studies. (+) low risk of bias; (?) unclear risk of bias; (-) high risk of bias.
